# Ultrafast energy transfer between lipid-linked chromophores and plant light-harvesting complex II†

**DOI:** 10.1039/d1cp01628h

**Published:** 2021-08-26

**Authors:** Ashley M. Hancock, Minjung Son, Muath Nairat, Tiejun Wei, Lars J. C. Jeuken, Christopher D. P. Duffy, Gabriela S. Schlau-Cohen, Peter G. Adams

**Affiliations:** School of Physics and Astronomy, University of Leeds Leeds LS2 9JT UK p.g.adams@leeds.ac.uk; Astbury Centre for Structural Molecular Biology, University of Leeds Leeds LS2 9JT UK; Department of Chemistry, Massachusetts Institute of Technology 77 Massachusetts Ave Cambridge MA 02139 USA gssc@mit.edu; School of Biological and Chemical Sciences, Queen Mary University of London Mile End Road London E1 4NS UK; Faculty of Biological Sciences, University of Leeds Leeds LS2 9JT UK; Leiden Institute of Chemistry, Leiden University 2300 RA Leiden The Netherlands

## Abstract

Light-Harvesting Complex II (LHCII) is a membrane protein found in plant chloroplasts that has the crucial role of absorbing solar energy and subsequently performing excitation energy transfer to the reaction centre subunits of Photosystem II. LHCII provides strong absorption of blue and red light, however, it has minimal absorption in the green spectral region where solar irradiance is maximal. In a recent proof-of-principle study, we enhanced the absorption in this spectral range by developing a biohybrid system where LHCII proteins together with lipid-linked Texas Red (TR) chromophores were assembled into lipid membrane vesicles. The utility of these systems was limited by significant LHCII quenching due to protein–protein interactions and heterogeneous lipid structures. Here, we organise TR and LHCII into a lipid nanodisc, which provides a homogeneous, well-controlled platform to study the interactions between TR molecules and single LHCII complexes. Fluorescence spectroscopy determined that TR-to-LHCII energy transfer has an efficiency of at least 60%, resulting in a 262% enhancement of LHCII fluorescence in the 525–625 nm range, two-fold greater than in the previous system. Ultrafast transient absorption spectroscopy revealed two time constants of 3.7 and 128 ps for TR-to-LHCII energy transfer. Structural modelling and theoretical calculations indicate that these timescales correspond to TR–lipids that are loosely- or tightly-associated with the protein, respectively, with estimated TR-to-LHCII separations of ∼3.5 nm and ∼1 nm. Overall, we demonstrate that a nanodisc-based biohybrid system provides an idealised platform to explore the photophysical interactions between extrinsic chromophores and membrane proteins with potential applications in understanding more complex natural or artificial photosynthetic systems.

## Abstract 2

We characterize the photophysical interactions between lipid-linked chromophores and plant light-harvesting proteins incorporated into nanodiscs using optical spectroscopy, simulations and theoretical modelling.

## Graphical abstract

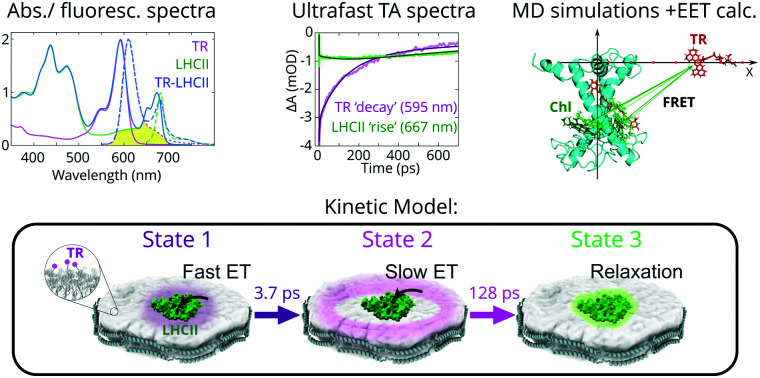

## 1 Introduction

Light-harvesting protein complexes (LHCs) are crucial for the effective absorption of solar energy in the first stages of photosynthesis. Antenna LHCs provide a large pool of precisely-organized pigments that facilitate multiple steps of excitation energy transfer and increase the rate of energy delivery to the reaction centre protein complexes, which perform a photochemical charge separation.^1^ A variety of LHCs exist across biology, but the majority are membrane proteins that have a specific 3-D polypeptide structure coordinating a network of pigments.^2–4^ Light-harvesting complex II (LHCII) is the major component of the peripheral antenna for the Photosystem II supercomplex in higher plants and green algae.^5^ Each monomeric subunit of LHCII contains a pigment network of 8 chlorophyll (Chl) *a*, 6 Chl *b* and 4 xanthophyll carotenoids.^6,7^ The pigments held within the protein scaffold form a dense array with inter-pigment distances as short as 0.97 nm, which enables the rapid and efficient transfer of excitation energy between pigments.^8,9^ While many of the excitation energy transfer steps within LHCII require a more complex description,^10^ transfer between pigments at distances above ∼1 nm can be accurately described with a straightforward perturbative treatment, Förster resonance energy transfer (FRET), as the hopping of localised excited states from one pigment to another.^3^ There has been great effort to understand the mechanisms underlying efficient transfer of excitation energy within and between LHCs and reaction centres in plants.^9,11–17^ There is also ongoing debate over the mechanism of photoprotective pathways within LHCII for the deliberate dissipation of energy under high-light conditions,^5,18–26^ including which specific pigments are involved, the nature of the pigment interactions, and the associated timescales.^27–29^ It is challenging to resolve the molecular details of proteins within their natural environment due to the complexity and dynamic nature of cells.^30^ Therefore, photosynthetic membrane proteins have been extensively studied in an isolated form, which allows the function and processes associated with specific proteins to be identified. Detergents can provide stable structures for isolating membrane proteins, and studies on LHCII isolated within detergent micelles have been highly revealing, showing the role of specific carotenoids,^12,17,31^ Chl–Chl interactions,^14,32–35^ how the proteins regulate light harvesting through aggregation,^36^ and the switching between different emissive states.^37–39^ However, detergents can often disrupt the structure of membrane proteins and therefore the ensued dynamics poorly mimic the natural biomembrane environment.^24,40,41^ In order to understand the physiologically relevant properties of LHCII, studies can be performed with the protein incorporated into a more native environment that includes a lipid bilayer.

Model membranes are an experimental system where membrane proteins are reconstituted into lipid bilayers at a defined protein-to-lipid ratio, which provides a native-like local lipid environment. The most common type of model membranes studied is spherical vesicles that are typically hundreds of nanometres in diameter, termed “proteoliposomes”. Proteoliposomes provide an appropriate 2-D confined expanse for membrane proteins, but can have uncertainties including the level of membrane curvature and the possibility of multiple (up/down) protein orientations within a single membrane.^42^ Protein–protein interactions can be studied within proteoliposomes, however, such interactions can sometimes cause undesirable effects. For example, in-membrane protein aggregation can cause changes to protein structure which may or may not be relevant to those found in nature.^43–48^ One alternative to proteoliposomes is to utilise disc-shaped model membranes, termed “lipid nanodiscs”, which consist of a lipid bilayer disc stabilised by amphipathic “belting proteins” (BP). Lipid nanodiscs are typically much smaller than liposomes with a diameter determined by the particular belting protein and stoichiometry of lipids used in sample formation. With these dimensions, architectures can be designed such that only one LHCII is incorporated per nanodisc, limiting the potential for protein–protein interactions to occur. Therefore, nanodiscs loaded with a single LHCII protein can be employed to disentangle the effects of protein–protein interactions (as with detergent micelles), but also allow the additional benefit of the lipid bilayer environment (as with proteoliposomes), providing the desired advantages of both systems.^24,47,49–51^

Synthetic chromophores can be interfaced with LHCs in order to provide additional pathways to inject excitation energy, where the extra chromophores are selected for their complementary spectral range or other photophysical properties of interest. Several studies have demonstrated successful energy transfer that resulted in an enhancement of the absorption range of the combined LHC–chromophore system as compared to native LHC. In these cases, energy transfer between synthetic and natural chromophores is non-radiative, analogous to transfer between natural chromophores in LHCs and can be generally described by simple FRET relationships. Typically, an approach of genetic manipulation or site-specific linker chemistry has been employed to covalently attach the external chromophore to the protein.^52–59^ In the best case, a hybrid chromophore–LHC system was reported with an effective absorption efficiency of up to 7 times relative to the unmodified LHC in the wavelength range where the protein's absorption is normally minimal.^55^ The efficiency of chromophore-to-protein energy transfer has been shown to surpass 85% for some hybrid systems with femto-to-picosecond timescales for energy transfer, similar to the that of inter-pigment energy transfer in natural LHCs.^54^ However, this covalent attachment strategy suffers from a limitation that it results in a system with low modularity, typically with one chromophore attached at a specific position to the LHC. Recently, we presented a flexible, non-covalent approach for interfacing synthetic chromophores to LHCs; a small, organic, lipid-linked “Texas Red” (TR) chromophore, which absorbs strongly in the region of minimal natural LHCII absorption, was co-assembled with plant LHCII into model lipid membranes.^60^ This approach exploited the spontaneous self-assembly of lipids to drive the system's formation and allowed a higher level of modularity than would be possible with chemical cross-linking or genetic modification. Specifically, the density of additional chromophores within the membrane system could be varied by simply changing the ratio of TR–lipids to normal lipids in the starting lipid mixture. Energy transfer was demonstrated from TR to LHCII with an efficiency up to 95%, and up to a three-fold enhancement of the fluorescence intensity of LHCII, when illuminating in the region of minimum natural absorption.^60^ The lipid-linked nature of TR allows it to be located within the lipid bilayer at a variable distance away from the protein, often 1–10 nm, comparable to the typical protein–protein distances found within natural light-harvesting membranes. Therefore, model lipid membranes containing extrinsic chromophores such as these could be a useful platform to probe ultrafast photophysical processes that have relevance to inter-LHC excitation energy transfer. Our previous investigation of TR and LHCII^60^ used proteoliposomes so that the interactions between TR molecules and many LHCII proteins could be observed; now, we introduce lipid nanodiscs as an elegant platform where interactions between TR and singular LHCII proteins can be observed and controlled using a confined membrane area. In the current study, we: (i) achieve a 262% enhancement of LHCII fluorescence in TR-LHCII nanodiscs as assessed with steady-state spectroscopy across the 525–625 nm range, (ii) observe rapid TR-to-LHCII energy transfer with ultrafast spectroscopy, and (iii) consider the molecular basis for the extracted timescales by generating a structural simulation of the system and performing theoretical calculations.

## 2 Results

### 2.1 Formation of model membrane samples

**Fig. 1:**
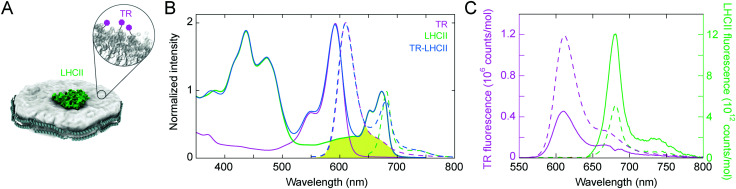
(A) Cartoon illustration of the nanodisc co-reconstituting TR–lipid and LHCII. TR–lipid is shown in light grey with TR molecules in purple (inset), LHCII is shown in green, and the belting protein (ApoE422K) is shown in dark grey. (B) Steady-state absorption (solid lines) and fluorescence emission spectra (dashed lines) of the TR liposomes (purple), the TR–LHCII nanodiscs (blue), and the LHCII nanodiscs (green). Spectra are normalised to either an intensity of 1.0 at the Chl *a* peak (675 nm) or 2.0 at the TR peak (590 nm), for clarity. The spectral overlap between the TR emission (energy donor) and the LHCII absorption (energy acceptor) is shown as the yellow shaded area. The excitation wavelengths used in fluorescence measurements are 540 nm (TR, TR–LHCII) and 630 nm (LHCII). (C) Steady-state fluorescence emission spectra acquired with excitation at 540 nm (2 nm bandwidth), focusing on the peak due to TR emission (in purple) or LHCII emission (in green). Spectra from the control samples containing only one component are shown as dashed lines, and the spectrum of the TR–LHCII nanodisc sample containing both components is shown as solid lines. For clarity, the spectrum of the TR–LHCII nanodisc was decomposed into its component TR and LHCII peaks (see ESI,† 2.6). For comparison, all spectra are shown as the “relative emission per mole”, where the TR peak is divided by the calculated molar TR concentration, and the LHCII peak is divided by the calculated molar LHCII concentration (see ESI,† 2.7).

Four components were required for our nanodisc samples: (i) the belting protein, which forms the enclosure of the disc and constrains its size, (ii) the LHCII membrane protein, which is to be incorporated into the lipid bilayer, (iii) the TR–lipid doped into the lipid bilayer at a low molar fraction (1.3%), (iv) the bulk, undoped, lipid, which forms the majority of the lipid bilayer. The stoichiometry of these components was chosen to incorporate a single LHCII protein into each nanodisc with close proximity to multiple TR molecules, as illustrated in Fig. 1A. We used a particular belting protein (ApoE422K) that can generate relatively large discs, expected to have diameters of 20–25 nm.^61^ This design provides a sufficient area around the LHCII to allow free lipid diffusion within the lipid bilayer annulus from the outer surface of a centrally-located LHCII to the disc edge. These dimensions allowed a range of donor–acceptor separations to be sampled as the protein and lipids diffuse laterally within the nanodisc, therefore, TR–LHCII separation distances both above and below the Förster radius (approx. 7 nm) can be achieved. The LHC used for all experiments was trimeric LHCII protein biochemically purified from spinach and solubilized in the detergent *n*-dodecyl-α-d-maltopyranoside (α-DDM; see ESI,† 2.1).^21^ The ApoE422K belting protein was overexpressed and purified from *E. coli* (see ESI,† 2.2).^24^ For all samples, the lipid mixture used was soy asolectin, which has been previously shown to provide a stable membrane environment for plant light-harvesting proteins reconstituted into nanodiscs.^24,50^ For samples containing TR, TR was included in the starting lipid mixture at a molar ratio of 1 : 75 relative to total lipids (1.3% mol/mol).

Three samples were prepared after several rounds of optimization of the relative ratios of the components to maximize nanodisc yields: (i) TR–LHCII nanodiscs as the main test sample of spectrally-enhanced LHCII; (ii) LHCII nanodiscs as the acceptor-only control sample; (iii) TR liposomes as the donor-only control sample. Note that nanodiscs were not required in the absence of LHCII, so, for sample (iii) liposomes were used for simplicity formed by following a typical sonication method.^21^ Chromophores linked to lipids have been shown to align in the same manner within both liposomes and nanodiscs,^62,63^ and thus we expect TR–lipid orientation to be similar in both samples.

The best yield of purified TR–LHCII nanodiscs at the target size was achieved with a starting mixture of a lipid : BP : LHCII ratio of 3000 : 12.5 : 1, where on average 6–7 copies of the belting protein associate to form a disc with ∼1500 lipids and 0 or 1 LHCII trimer (see ESI,† 1.2).^24^ Particle characterisation measurements were performed on nanodisc samples to determine the distribution of the disc sizes and to rule out the presence of any large protein aggregates. Transmission Electron Microscopy (TEM) analysis gave an average nanodisc diameter of 21 ± 7 nm (mean ± S.D.), in agreement with dynamic light scattering (DLS) measurements (see ESI,† 2.4). Importantly, neither TEM nor DLS indicated the presence of any large aggregates of proteins or lipids, suggesting that the vast majority of particles in the final preparation were individual nanodiscs. Therefore, the predominant TR–LHCII interactions were considered to be between multiple TR moieties and single LHCII proteins reconstituted into discs.

### 2.2 Steady-state spectroscopy

To determine the concentrations of LHCII and TR achieved within the membrane samples and to assess any spectral shifts, steady-state absorption spectroscopy was performed (Fig. 1B, solid lines). The absorption peaks representing LHCII at 400–500 nm and 630–700 nm are identical both in the presence and absence of TR, suggesting that TR has no direct effect on the pigment structure within LHCII (blue *vs.* green solid lines in Fig. 1B). These peaks are very similar to the absorption spectrum for isolated LHCII in detergent micelles (see ESI,† 2.5), indicating that the structure and pigment composition of LHCII are maintained within the lipid environment of the nanodisc. The TR peak at 590 nm has the expected shape and position in its samples, whether it is within nanodiscs or liposomes. Analysis of the absorption spectra allowed the absolute concentration of each component to be calculated using known absorption coefficients after spectral decomposition to isolate the signal from each component (see ESI,† 2.6 and 2.7). The TR–LHCII nanodisc sample had an estimated lipid : TR : LHCII ratio of 2870 : 38.3 : 1 (with the assumption that the ratio of TR–lipid to normal lipids is not altered during the assembly process). These values are consistent with predicted ratios based on predicted nanodisc geometry, confirming that sample preparation was successful.

TR-to-LHCII energy transfer was assessed *via* steady-state fluorescence spectroscopy with selective excitation of TR at 540 nm, where LHCII has minimal absorption. As shown in Fig. 1C, the fluorescence intensity of TR was significantly reduced in the membrane with LHCII compared to those without (solid *vs.* dashed purple lines). This quenched emission from TR (the energy donor) was quantified as 38% relative to the TR-only control sample, therefore, the efficiency of resonance energy transfer from TR-to-LHCII is estimated as 62% according to simple Förster theory interpretation (see ESI,† 2.8).^60^ This value for transfer efficiency agrees with independent measurements using a comparison between LHCII fluorescence excitation spectra and “absorptance” (1 − transmission) spectra, which suggested an efficiency of ∼65% (see ESI,† 2.9). This observed efficiency is lower than we expected (compared to our previous findings on TR–LHCII proteoliposomes^60^) and is attributed to the presence of unloaded nanodiscs in the sample, which contain DOPC and TR lipids but no LHCII. TR fluorescence in these discs will be unquenched, and thus skew the overall energy transfer efficiency calculated towards a lower value. The true value for TR-to-LHCII energy transfer efficiency in nanodiscs containing both LHCII and TR can be estimated as >90% when the fraction of the unloaded nanodiscs is taken into account (see ESI,† 2.10). So far, we have only considered direct transfers from the nearest TR molecule to LHCII, however, TR molecules that are next-nearest (and beyond) could also indirectly transfer excitation energy to LHCII *via* intermediary TR molecules. In fact, we expect that a series of TR-to-TR transfers could occur, which would increase the overall TR-to-LHCII transfer efficiency in the system. Whilst we cannot access this experimentally due to the indistinguishability of donor- and acceptor-specific spectral features, we can estimate the TR–TR couplings theoretically and calculate that the TR–TR energy transfer efficiency would be ∼92% at the average TR–TR separation distance of 4.0 nm (see ESI,† 2.11). Thus, it is likely that a series of transfers will occur and that the overall measured transfer time will be dominated by the nearest-neighbour TR to an LHCII.

LHCII fluorescence can also be assessed to show the direct effect of TR on LHCII's photophysical properties. Probing isolated LHCII with 540 nm excitation leads to a minimal level of fluorescence, whereas, in the combined system, an enhancement of LHCII fluorescence would be expected if additional energy is transferred from TR donors to LHCII acceptors. The effective absorption strength of LHCII in nanodiscs is enhanced by 262% in the 525–625 nm range, which equates to an increase of 29.7% across our entire measurement range of 380–680 nm, which also represents the entire spectrum of visible light (see solid *vs.* dashed green lines in Fig. 1C and calculations in ESI,† 2.12). When considering a natural excitation source, such as the AM1.5 solar spectrum, this enhancement across the full visible range increases to 32.6% due to the strong absorption of TR at wavelengths of high solar irradiance (as shown in ESI,† 2.13).

### 2.3 Time-resolved fluorescence

**Fig. 2:**
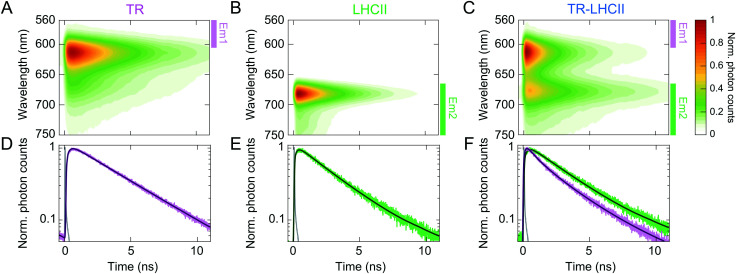
Normalised TRFS of TR liposomes (A), LHCII nanodiscs (B), and TR–LHCII nanodiscs (C). (D)–(F) Fitted decay traces of the TRFS shown in (A)–(C). The wavelength ranges over which the fluorescence signal was integrated are labelled in (A)–(C) with coloured bars (excitation at 550 nm, Em1: 560–600 nm, Em2: 665–780 nm, see ESI,† 2.14). The instrument response function (IRF) is shown in grey in (D)–(F).

In order to determine the timescale of FRET from TR to LHCII, time-resolved fluorescence spectra (TRFS) of all three samples were measured by selectively exciting the TR donor and simultaneously detecting the fluorescence dynamics across the entire emission range of each sample (Fig. 2 and ESI,† 2.14). The TRFS of TR liposome (see Fig. 2A) and LHCII nanodisc (Fig. 2B) exhibit emission maxima at 614 nm (TR) and 682 nm (LHCII), respectively, accompanied by vibronic bands at 673 nm (TR) and 735 nm (LHCII), in accordance with their steady-state fluorescence spectra. The fluorescence decay kinetics do not show dependence on the emission wavelength for either sample (see ESI,† 2.15). The fluorescence decay of TR liposome was fitted to a monoexponential decay with a time constant of 3.94 ± 0.20 ns (Fig. 2D and Table 1), in agreement with the value reported in the literature of ∼4 ns for isolated TR chromophores.^64^ The LHCII nanodisc showed a biexponential decay profile, resulting in an average fluorescence lifetime (〈*τ*_fl_〉) of 2.68 ± 0.06 ns (Fig. 2E), which represents a moderate degree of quenching (∼30%) as compared to the lifetime reported for LHCII isolated in detergent micelles of ∼4 ns.^21,40,43^ Such quenching of LHCII fluorescence in nanodiscs has been reported previously, and attributed to conformational changes in LHCII induced by the local membrane environment.^24,25,50^

The TRFS of the TR–LHCII nanodisc (Fig. 2C) appears as the combination of those of TR and LHCII, with emission maxima at 614 nm and 677 nm. The first band originates almost exclusively from TR fluorescence, and the second band contains mixed contributions from both the (0–1) vibronic band of TR fluorescence and the (0–0) band of LHCII fluorescence. Unlike for the TR liposome or LHCII nanodisc, the fluorescence decay profile of the TR–LHCII nanodisc shows dependence on the emission wavelength, due to the presence of both TR and LHCII. We monitored two emission ranges, labelled Em1 and Em2 in Fig. 2, which originate from predominantly TR fluorescence and LHCII fluorescence, respectively. In the emission range that reports the decay of the TR fluorescence (Em1), we observe a 68% decrease in the 〈*τ*_fl_〉 of the TR–LHCII nanodiscs compared to that of the TR liposome, from 3.94 ± 0.20 ns to 1.28 ± 0.03 ns (Table 1). This corresponds to a FRET efficiency of 68%, in good agreement with the 62% efficiency calculated from the analysis of the steady-state fluorescence presented in Section 2.2. Similarly to the calculations using steady-state data, this value is likely to be an underestimate of the true energy transfer efficiency (ETE) in those nanodiscs containing both TR and LHCII (estimated at >90%, see ESI,† 2.10) due to the presence of TR in nanodiscs where LHCII is absent.

**Table 1:** Fit parameters from time-resolved fluorescence measurements^a^

	*A*_1_ (%)	*τ*_1_ (ns)	*A*_2_ (%)	*τ*_2_ (ns)	〈*τ*_fl_〉 (ns)
TR (Em1)	—	—	100	3.94 ± 0.20	3.94 ± 0.20
LHCII (Em2)	10.2 ± 0.4	0.33 ± 0.04	89.8 ± 0.4	2.95 ± 0.07	2.68 ± 0.06
TR–LHCII (Em1)	58.8 ± 0.4	0.32 ± 0.02	41.2 ± 0.4	2.64 ± 0.06	1.28 ± 0.03
TR–LHCII (Em2)	66.4 ± 0.6	2.60 ± 0.20	33.6 ± 0.6	3.92 ± 0.08	3.17 ± 0.13

a *A*: percent amplitude, *τ*: time constant, 〈*τ*_fl_〉: amplitude-weighted average fluorescence lifetime (〈*τ*_fl_〉 = *A*_1_*τ*_1_/100 + *A*_2_*τ*_2_/100). The errors shown after the ± symbol are the 95% confidence intervals of the fit.

The fluorescence decay in the red emission range (Em2) is best fitted to a biexponential decay profile, resulting in a longer 〈*τ*_fl_〉 of 3.17 ± 0.13 ns compared to that of LHCII without the TR donor. This apparent lengthening of 〈*τ*_fl_〉 is attributed to the aforementioned convolution of the LHCII fluorescence with the (0–1) vibronic band of TR, which has a longer lifetime as well as a higher fluorescence quantum yield than those of LHCII, and thus slows the detected fluorescence decay.

### 2.4 Transient absorption

The ultrafast timescale of energy transfer from TR to LHCII was measured with femtosecond transient absorption (TA) spectroscopy. The pump laser spectrum was tuned to preferentially excite the absorption band of the TR donor from 500 to 600 nm. A broadband probe pulse spanning both the TR and LHCII transitions was employed (see ESI,† 2.16). To fit the TA data, global analysis was performed, which can disentangle the multiple competing processes and identify the specific time constants for the temporal dynamics and their associated spectral signatures.^65^

**Fig. 3:**
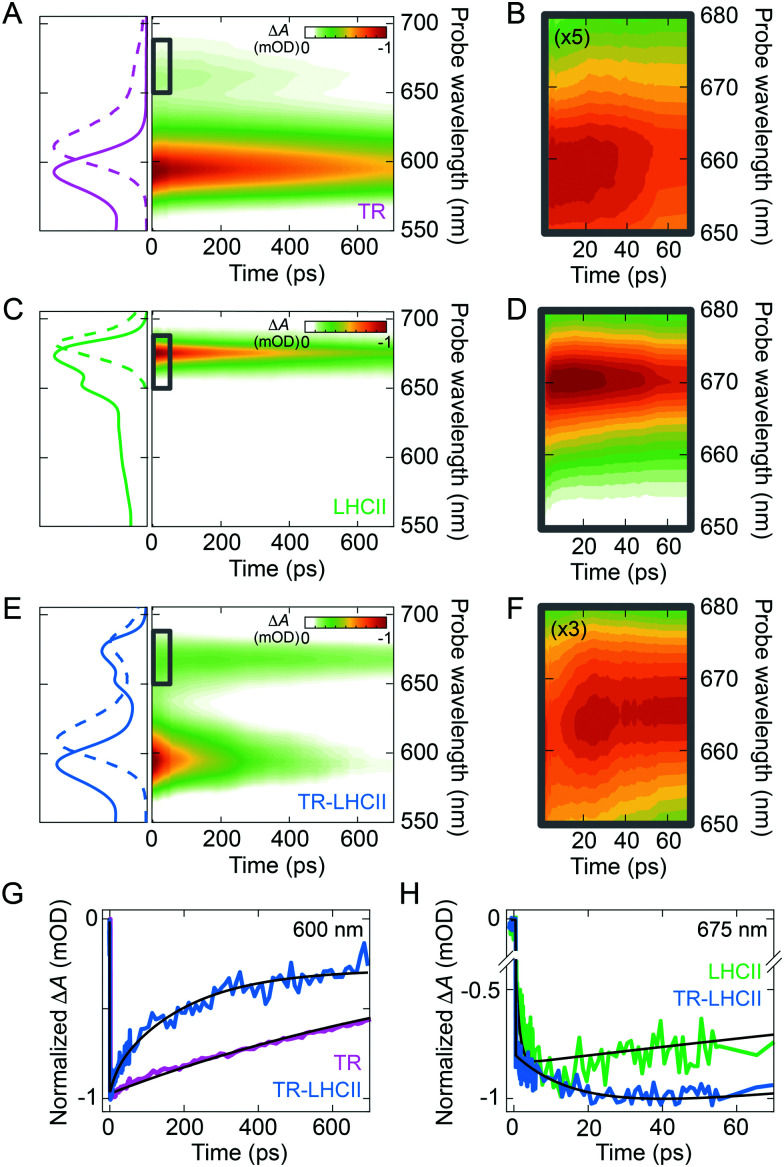
Normalised TA maps of TR liposome (A and B), LHCII nanodisc (C and D), and TR–LHCII nanodisc (E and F). Steady-state absorption (solid lines) and fluorescence (dashed lines) spectra of each sample are shown on the left. (B, D and F) are zoomed-in regions showing the Chl Q_*y*_ GSB/SE range for the initial 70 ps (grey boxes in (A, C and E)). The colour-scale in the maps in (B) and (F) are multiplied by a factor of 5 and 3, respectively, for clarity. (G) Normalised fitted time traces at 600 nm demonstrating the difference in TR excited-state decay. (H) Normalized fitted time traces at 675 nm demonstrating the rise in the population of LHCII excited state (initial 70 ps). The black lines in (G) and (H) show the fits discussed in the text.

To benchmark the excited-state dynamics of the TR donor and the LHCII acceptor in the absence of their FRET partner, we measured the TA of TR liposome and LHCII nanodisc. The TA map of TR liposome (Fig. 3A and B) shows ground-state bleach (GSB) and stimulated emission (SE) with a main peak at 593 nm and a minor peak at ∼658 nm, in accordance with the vibronic band structure seen in the steady-state absorption and fluorescence spectra. The main peak exhibits a mono-exponential decay with a time constant of 1.17 ± 0.02 ns (see ESI,† 2.17). The TA map of LHCII nanodisc (Fig. 3C and D) shows an intense band peaking at ∼675 nm, which is the GSB/SE of the Chl *a* Q_*y*_ state. The TA signal at shorter wavelengths (<650 nm) is very weak due to the low oscillator strength of the higher vibronic states as well as overlapping contributions from the negative GSB/SE and positive excited-state absorption (ESA).^12,66^ The LHCII GSB/SE decay was fitted with a single time constant of 903 ± 8 ps (see ESI,† 2.17). For both TR and LHCII, the fitted time constant from the TA is shorter than the lifetime extracted from the TRFS (Table 1) because of the limited temporal window of our TA measurement (0–700 ps).

**Fig. 4:**
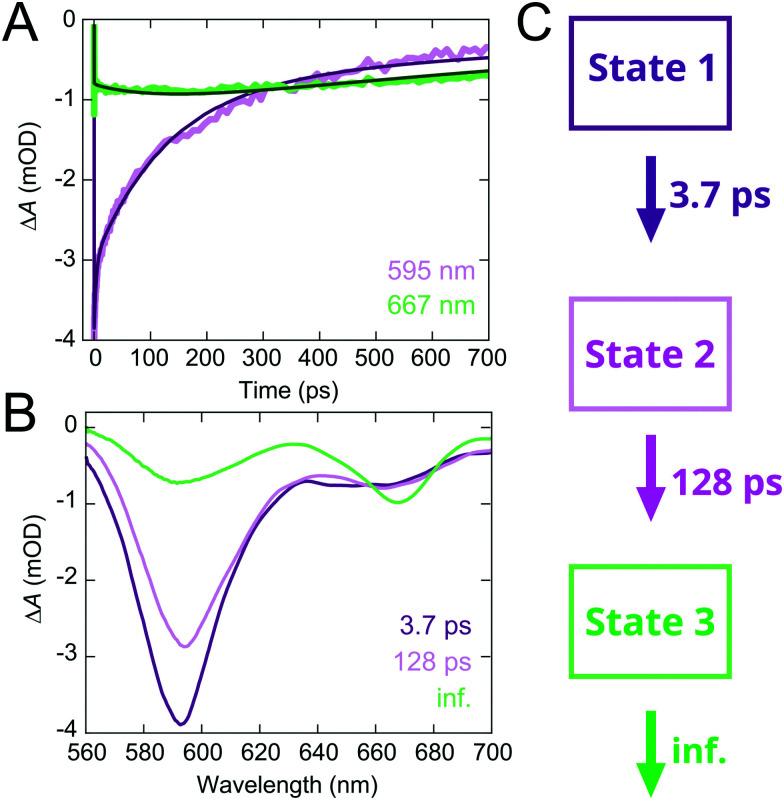
Global analysis of the TA data of the TR–LHCII nanodiscs. (A) Global fit of the TA decay traces at 595 nm (purple) and 667 nm (green). (B) EADS of the three components shown in (A): EADS1 (3.7 ps), EADS2 (128 ps), EADS3 (inf.) (C) Kinetic model of the dynamics observed in TR–LHCII nanodisc with the associated time constants obtained from global analysis.

The TA map of the TR–LHCII nanodisc (Fig. 3E and F) exhibits spectral features of both TR and LHCII, *i.e.*, the GSB/SE from TR centred at 593 nm and that from LHCII Chl *a* centred at ∼665 nm. Due to the weak absorption of LHCII in the pump wavelength range that partly overlaps with TR absorption, assigned to higher vibronic levels of the Q_*x*_ and Q_*y*_ states of the Chls,^67,68^ a small percentage (∼20%) of the excited state measured is due to direct absorption by the Chls. These higher vibronic states undergo rapid internal conversion within the vibronic manifold on a ∼200 fs timescale to the lowest vibronic level of Q_*y*_.^69–71^ Comparison of the decay of the donor TR peak with and without the acceptor LHCII demonstrates significant acceleration of the TR decay in the presence of LHCII (Fig. 3G). Global analysis reveals that TR excited states have a much faster biexponential decay with time constants of 3.7 ± 0.1 ps and 128 ± 1 ps in the presence of LHCII (compared to the 1.17 ns time constant in the absence of LHCII). These excited states and their associated time constants are shown as a kinetic model in Fig. 4C (further discussed in Section 2.5). In addition, we observe a rise of the LHCII GSB/SE band on a similar timescale (Fig. 3H). The TA data at wavelengths selective for the LHCII and TR components in the TR–LHCII nanodiscs are shown with the fits from global analysis in Fig. 4A. The concomitant decay of the TR and rise of the LHCII population in the TR–LHCII sample clearly indicate the presence of energy transfer from TR to LHCII on a picosecond timescale.

We also examined the spectral evolution using the evolution-associated difference spectra (EADS) and the decay-associated difference spectra (DADS), which show the spectral signature and the spectral change, respectively, for the process described by each time constant. The EADS associated with each time constant for the TR–LHCII nanodiscs are shown in Fig. 4B. The first two EADS, which are associated with time constants of 3.7 and 128 ps (EADS1 and EADS2, respectively), are dominated by the TR peak at 593 nm. The negative sign of this peak in the DADS in ESI,† Fig. S14 (DADS1, DADS2) means that these two constants represent a biexponential decay of the TR population. Furthermore, these EADS show a growth in the TA signal at longer wavelengths with predominantly LHCII contribution (>650 nm), as demonstrated by the positive sign of the feature in the DADS in ESI,† Fig. S14. While this feature is clearly observed in DADS2, it has a small amplitude in DADS1, likely due to overlapping decay of the TR vibronic shoulder (Fig. 3A). However, the emergence of the fast 3.7 ps TR dynamics is exclusive to the TR–LHCII nanodiscs (see ESI,† 2.18), supporting the assignment of this component to energy transfer. The 3.7 ps component could not be applied to TR-only liposomes or LHCII-only nanodiscs, further supporting the assignment of this component to a TR–LHCII energy transfer pathway (see ESI,† 2.19). Thus, we assign these two components to the timescales of energy transfer from TR to LHCII. The biexponential decay kinetics observed suggests that the energy transfer is heterogeneous in nature, as may be expected from a distribution of donor–acceptor distances within the nanodisc due to the large number of positions that TR–lipids can have within the expanse of lipid bilayer (further discussion in Section 3.1). It should be noted that these extracted timescales are likely averages of each subpopulation and not exact values. The final, long-lived EADS (inf.) represents the decay processes that are significantly slower than the time window of the TA measurements (EADS3 in Fig. 4B). It is likely that this EADS represents the combination of the nanosecond decay of LHCII and the nanosecond decay of a sub-population of TR within lipid nanodiscs that are too far from LHCII to interact with. Although the data was well fitted with a biexponential decay, a distributed exponential could also describe the kinetics due to the observed heterogeneity within the TR–LHCII sample.

While the sequential model described here provides a good fit to the data, a branched model can also be employed (see ESI,† 2.20). In this case, similar time constants (7 ps and 239 ps) were extracted with peaks indicative of TR-to-LHCII energy transfer, although the amplitudes were more challenging to rationalize. For this reason, we focus on the sequential model, although the system likely contains a mixture of multiple energy transfer pathways including both sequential and parallel steps. For example, we would expect TR–TR energy transfer to occur sequentially before TR–LHCII energy transfer, and both slow and fast TR–LHCII energy transfer to occur in parallel. The time constants obtained using the branched model were found to be compatible with those extracted from the TCSPC data (see ESI,† 2.20).

### 2.5 Molecular dynamics simulations and energy transfer rates

**Fig. 5:**
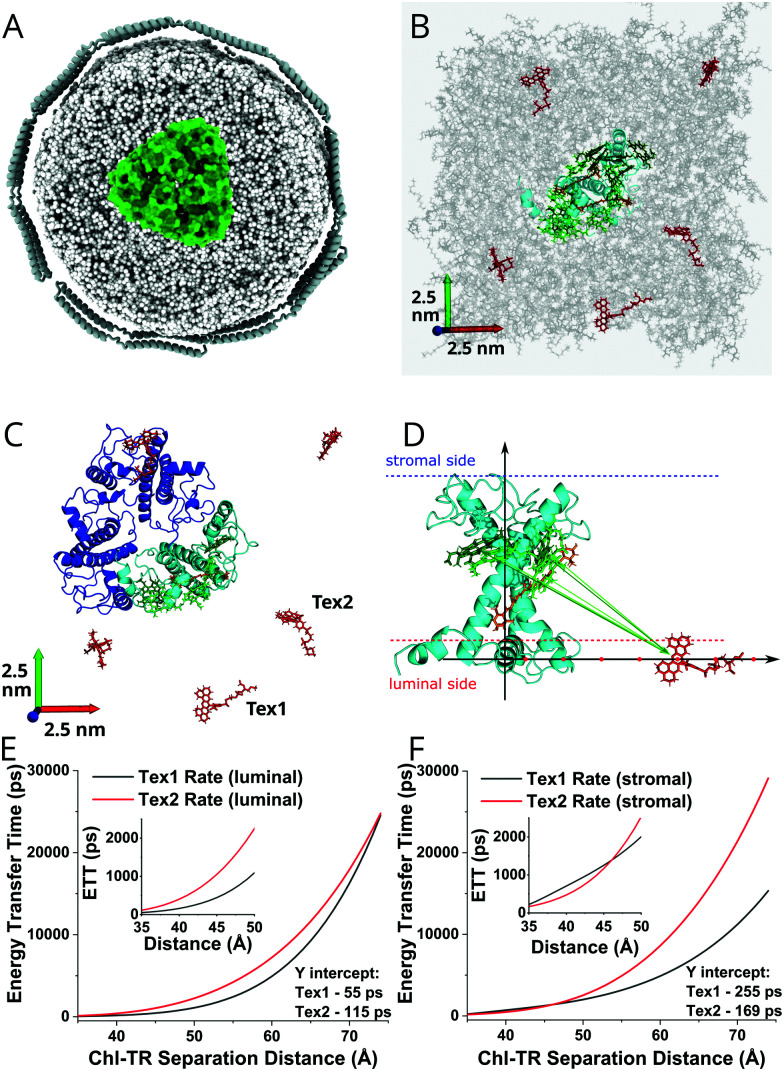
(A) Cartoon of our lipid nanodisc system (top-down view) showing an LHCII trimer (green), surrounded by lipids (light grey), enclosed within the belting proteins (dark grey). This cartoon is approximately to scale. (B) Molecular dynamics model of the TR–LHCII-lipid system (top-down view) at the start of the simulation, *t* = 0. Shown are approximately 500 DOPC lipids (grey), 5 Texas Red (red), one LHCII monomer (polypeptide in cyan, Chls in green, carotenoids in orange). Only the most relevant pigments are displayed for clarity. (C) Representation of the system from (B), except overlaid with the position of a possible LHCII trimer. The two TR molecules of interest that were chosen for further analysis are noted (Tex1 and Tex2). Only the protein backbone and pigments at the extremity of the protein, which we expect to interact with TR, are shown for clarity. (D) Side-on view of the structural model demonstrating the simulated change in lateral position of the TR molecule relative to LHCII incorporated into the lipid bilayer. (E and F) Graphs showing the TR-to-Chl transfer times (*k*^−1^) as a function of separation distance as the TR position is varied as shown in (D), for the Chl*a*612-to-Tex1 pair (black line) or the Chl*a*612-to-Tex2 pair (red line). This was computed from the resonance couplings and the spectral overlaps for all likely transitions, following Förster theory as detailed in ESI,† 2.24. (E) and (F) show the transfer rates calculated from TR located on the luminal and stromal sides of the membrane, respectively. The inset graphs magnify the low-distance regime for increased clarity.

To investigate the structural context for the energy transfer timescales observed, we performed molecular dynamics (MD) simulations and energy transfer calculations on the TR–LHCII nanodiscs. The simplest possible arrangement for a TR–LHCII nanodisc is a single LHCII trimer embedded centrally within the lipid nanodisc, as shown in the cartoon in Fig. 5A. Of course, this simple picture is woefully imprecise and does not properly consider structural clashes and other interactions and fails to consider any protein/lipid dynamics within the system. In order to get a more accurate picture of the interactions and timescales occurring within TR–LHCII nanodiscs, we performed a set of short MD simulations. A bilayer membrane comprised of 500 1,2-dioleoyl-*sn-glycero*-3-phosphocholine (DOPC) lipids was generated with both width and length of 125 Å. An LHCII monomer, structure adopted from Protein Data Bank (PDB) 1rwt,^6^ was inserted into the bilayer^72^ with sterically clashed lipids carefully removed (see ESI,† 2.21 for more detailed information). Note, a monomer was chosen for computational simplicity as in other previous MD simulations of LHCII.^73^ In accordance with the experimental concentration, five TR-tagged lipids were placed randomly in the membrane. The generated system is shown in Fig. 5B. Initially, all five were positioned at what, in the native membrane, would be the stromal side of the protein, although both orientations are likely sampled in the nanodisc. To account for this, once the MD trajectories had been computed a new set of trajectories were generated by simply flipping the five TR to the “luminal” side of the membrane. This avoided the cost of repeating the MD simulations and is entirely reasonable given the large distance between the TR and LHCII.

The system was first equilibrated over 20 ns before five independent simulations were performed of 60 ns duration each. The resulting trajectories were analysed in order to reveal the motion of the protein, lipids and TR. The calculated diffusion constant for the non-tagged lipid was found to be 8.4 μm^2^ s^−1^ (see ESI,† 2.22), in good agreement with previously published calculations^74^ and within the experimentally reported range of 5–14 μm^2^ s^−1^.^75^ The TR-tagged lipid tail exhibited slower diffusion (4.5 μm^2^ s^−1^), reflecting the hydrodynamic drag of the TR. Due to the symmetry we expect these values to be identical when TR is located on the luminal side of the membrane.

It would take a prohibitively long simulation to properly sample the diffusion of the TR–lipid over the long distances required to generate close interactions with LHCII, so this was not tested computationally here. Instead, these simulations provide the structural model that will be used as a physically reasonable geometry for theoretical calculations of energy transfer described below. Within the LHCII monomer, the pigment cofactors largely retain their original positions (see ESI,† 2.23). Of the five TR–lipid molecules, we chose to look more closely at the two (labelled Tex1 and Tex2 in Fig. 5C) that would be closest to the outer edge of LHCII if our monomer was part of a trimer as in the experimental system. The closest TR molecules are expected to transfer the most energy to LHCII. The orientation of these two TR were quantified in terms of the angle between their transition dipole moment and the normal of the membrane. One maintained an angle of 20–40° to the membrane normal (*i.e.*, relative to the average lipid orientation), which is consistent with earlier simulations,^62^ while the other adopted a nearly perpendicular orientation. We then calculated the resonance couplings between these two TR and each of the membrane-exposed Chls within LHCII (Chl *a*610–611–612, Chl *b*601 and Chl *b*608) following the theory described in ESI,† 2.24. Because the distance between TR and the Chls is larger than the physical extent of the molecules themselves, the point dipole approximation was used.^76^ For the stromal TR, the couplings are small, which is to be expected given the large TR–Chl distances. For the Q_*y*_ transitions, the largest coupling is between Tex2 and Chl *a*612 with |*J*| = 0.15 meV, although it fluctuates between 0.1–0.3 meV. For the Q_*x*_ band, the strongest is between Tex2 and Chl *a*610 with |*J*| = 0.04 meV, fluctuating between 0.01–0.1 meV. For comparison, within LHCII the typical Chl–Chl separation distances are ∼1–2 nm and the coupling ranges from 1–10 meV. When we look at our reconstructed luminal TRs the relative couplings are different and overall a little stronger, which is to be expected given that the surface-exposed Chls are closer to the luminal side of the protein. For Q_*y*_, the strongest is between Tex1–Chl *a*611 (|*J*| = 0.22 meV), but Tex1 also couples to Chl *a*612 and Chl *b*608 (|*J*| = 0.19 meV). For Q_*x*_, the strongest pair is Tex2–Chl *a*610 with |*J*| = 0.13 meV.

Using the resonance couplings, it is possible to compute first-order (Förster) rate constants for energy transfer between TR and the Q_*y*_/Q_*x*_ states of the five Chls (see ESI,† 2.24). These rates also depend on the normalised spectral overlap between donor and acceptor transitions, which was calculated using an experimentally-derived TR fluorescence spectrum^60^ while the Chl Q_*y*_/Q_*x*_ transitions were assigned Gaussian lineshapes and computed using a standard model of the system-bath interaction.^77^ Strictly, the acceptor states in LHCII will be a mixture of single-molecule and excitonic states; however, since excitonic interactions in LHCII do not appear to produce significant peak shifts or redistribution of oscillator strengths,^77^ they can be neglected without qualitatively altering the calculated rates. The total rate was then defined as the sum of all rates associated with a single TR. Due to the relatively large distances between the randomly placed TR molecules and LHCII, transfer rates are slow. For the stromal TR we get *k*_total_^−1^ = 4.4 ns and *k*_total_^−1^ = 1.9 ns for Tex1 and Tex2, respectively, with the difference simply reflecting the fact that Tex2 is a little closer to LHCII. When we consider the luminal TR transfer times are noticeably faster, with *k*_total_^−1^ = 1.1 ns and *k*_total_^−1^ = 0.8 ns, respectively. Despite this difference, all of the transfer times are far longer than those observed experimentally. We must therefore consider a system with shorter TR–LHCII distances in order to explain the energy transfer timescale observed in our spectroscopy experiments.

The distance dependence of these rates was then probed by manually moving the two chosen TR molecules along a vector connecting the lateral position of the tagged lipid and the centre of mass of the LHCII monomer (Fig. 5D). The rates of energy transfer were calculated assuming a time-averaged transition dipole moment for the TR molecules (see ESI,† 2.25). The transfer-distance curves for the two stromal TR (Tex 1 and Tex2) are shown in Fig. 5F. The smallest distances assessed (∼35 Å) represent the onset of clashes between the LHCII and TR–DOPC structures. At this distance, the total transfer times are *k*_total_^−1^ = 225 and 169 ps for Tex1 and Tex2, respectively. For the luminal TR, these values are 55 and 115 ps (see Fig. 5E). The fastest transfer time is still an order of magnitude slower than the 3.7 ps component observed in the TA kinetics. A possible reason for this is that, at these close distances, the point dipole approximation will break down and couplings with Chls buried within LHCII may become significant, meaning our calculated transfer times are likely overestimates. Even at these close distances, the TR chromophores lie on the outside of the membrane (Fig. 5D), which precludes the very close contacts needed for ∼3.7 ps transfer times. However, such short timescales could be achieved in theory if LHCII was closely surrounded by several TR-tagged lipids or if TR molecules relocated into the hydrophobic core of the lipid bilayer (closer to the *z*-position of the Chls).

## 3 Discussion

In this study, we generated lipid nanodiscs containing the plant LHCII antenna complex and synthetic TR chromophores, and showed that they are energetically coupled. Biochemical and steady-state absorption data determined that the assembly procedure successfully incorporated LHCII and TR together into nanodiscs (Fig. 1A, B and ESI,† Tables S1, S2). Steady-state and time-resolved fluorescence spectroscopy provided evidence that resonance energy transfer occurred from TR to LHCII with an overall efficiency of at least 60% (Fig. 1C and 2). Ultrafast time-resolved absorption data of TR–LHCII nanodiscs showed that the excited-state population of TR decays on a sub-100 ps timescale with a concomitant rise of the LHCII excited state, establishing the presence of rapid energy transfer (Fig. 3 and 4). Computer modelling of the lipid–TR–LHCII assembly then allowed us to assess the structural dynamics and possible pathways for energy transfer (Fig. 5). Two distinct timescales of TR-to-LHCII energy transfer were determined, ∼3.7 ps and ∼128 ps, which we assign to different TR–LHCII separation distances. To provide context: a 1–10 ps timescale for TR-to-LHCII energy transfer is significantly slower than the intra-protein Chl–Chl energy transfer, which is known to occur at the tens of femtoseconds to single-digit picoseconds timescales,^8,14^ and is more comparable to that of inter-protein energy transfer between neighbouring LHCs in natural thylakoid membranes.^78,79^ We discuss the implications of our results below.

### 3.1 Molecular-level interpretations of experimental and theoretical data

**Fig. 6:**
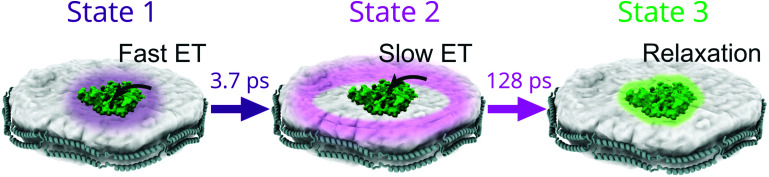
Cartoons illustrating the proposed scheme of TR-to-LHCII energy transfer derived from our combined interpretation of the TA and FRET modelling data. The excited-state population evolves over time from State 1 to State 2 as TR molecules that are tightly coupled to LHCII decay with a fast timescale (purple), and from State 2 to State 3 as TR molecules that are loosely coupled to LHCII decay with a slower timescale (pink). From State 3, excited Chls within LHCII will relax to the ground state with a timescale exceeding the temporal window of our TA measurement (green).

By consideration of both our experimental and computational data we can describe the likely molecular nature of the TR–LHCII interaction. The nanodisc system with TR chromophores laterally diffusing around the reconstituted LHCII protein appears to have two distinct energy transfer processes according to the TA data: there is one pathway with a relatively fast 3.7 ps time constant and another with a slower, 128 ps time constant. These likely correspond to a sub-population of TR that is tightly coupled (fast ET – State 1) to LHCII and a sub-population of TR that is more loosely coupled to LHCII (slow ET – State 2) as illustrated in Fig. 6. In the global analysis of the TA data used to identify these time constants, the best fit to the data is achieved with a sequential model (see Fig. 4C): upon initial photoexcitation of all TR molecules, the TR sub-population that is tightly coupled to LHCII (fast ET - State 1) transfers energy to LHCII on a 3.7 ps timescale. This fast step is followed by a slower (128 ps) energy transfer step, where the loosely-coupled sub-population of TR (slow ET – State 2) transfers energy to LHCII. The presence of two separate TR-to-LHCII energy transfer components suggests that the tightly- and loosely-coupled sub-populations are independent within the measured ensemble and thus the sequential model shown in Fig. 4C and 6 reflects temporal, rather than causal, separation of the dynamics. That is, on the timescale of energy transfer the two sub-populations are static, where any interconversion is likely through lipid diffusion on the microsecond timescale.^80^ Finally, the last time constant (inf.) is attributed to the relaxation of the LHCII excited state as well as TR molecules that are too far to interact with LHCII and therefore not involved in energy transfer. The analysis of our data cannot specifically distinguish whether the sequential model describes a two-step energy transfer pathway as the spectra of all the TR are the same. Instead, the sequential evolution of our system can be interpreted as sequential changes to the relative populations of excited TR and excited LHCII. However, this model is an approximation, as the actual energy transfer pathways likely involve a series of heterogeneous energy transfer steps from the TR to LHCII and amongst the TR, although the latter cannot be resolved in our measurements due to a lack of associated spectral changes. Despite the heterogeneous nature of the energy transfer pathways, the spectroscopic data show an overall highly efficient funnelling of energy from the TR to LHCII, thereby increasing its light-harvesting capacity.

The observation of a ∼4 ps transfer rate in TA data suggests that the TR and Chl of LHCII must be within ∼22.5 Å separation as energy transfer rates of under 20 ps could not be achieved in our theoretical simulations with single TR molecules even at the closest TR–LHCII distances of 35 Å (see Fig. 5E and F). This implies that an additional TR–LHCII interaction must be taking place. We speculate on a few possible molecular origins for this interaction. The first possibility would be a direct electrostatic attractive interaction between the TR moiety and the surface of the LHCII. This could be mediated by the negative charge of a sulfonate group which is intrinsic to the structure of TR molecule^81^ interacting with amino acids protruding from LHCII, either positively-charged residues on the stromal surface, or amphiphilic helices on the luminal surface.^7^ A second possibility is hydrophobic-hydrophobic interactions between the membrane-embedded portion of LHCII and the fatty acyl tails of the lipid attached to TR. Our MD simulations did not test for these direct interactions due to computational time limitations, but this seems feasible as lipid–protein interactions often occur between lipids and membrane proteins, including in LHCII and other thylakoid proteins.^6,82^ If such attractive interactions occur, then they would tend to concentrate the TR around the protein to provide a biased distribution with much shorter average TR-to-LHCII distances than if TR molecules were randomly distributed in the membrane. If multiple TR molecules cluster together in close proximity to the LHCII, potentially due to one of the two interactions suggested above, excitonic interactions between coupled chromophores would increase the rate of energy transfer to faster than 5 ps, consistent with our experimental data (due to the additive nature of transfer rates when multiple donors are interacting with a single acceptor). Although TR is negatively charged, attractive interactions could be mediated *via* stacking of the cyclic xanthene ring of TR.^81^ Another possibility is that the TR moiety does not always locate outside of the lipid bilayer but can actually flip into the hydrophobic portion of the lipid bilayer, significantly decreasing the possible TR–Chl separation and accounting for the faster-than-simulated rate of energy transfer.^62^ While this is not supported by our MD simulations, which found that all TR remained outside the bilayer in various orientations, we cannot rule out the possibility of the TR entering the bilayer as suggested previously.^62^ The more loosely coupled population of TR represented by the 128 ps time constant in TA data is more easily explained by TR located at approximately 30–40 Å from the nearest Chl within LHCII (from inspection of the inset in Fig. 5E), suggesting a TR molecule that has a few intervening lipids between itself and the outer external surface of LHCII.

The significance of our structural interpretations on our TR–LHCII system is that they provide hints towards the strategic design^83^ of favourable interactions between pigments and proteins to enable tight coupling and high rates of energy transfer. We envision that a promising future direction in studies of synthetic/biological hybrid systems would be to construct systems bearing chromophores that promote direct attractions either between the hydrophilic external surface of the LHC protein and charged pigments or between the hydrophobic membrane subunits of LHCs and neutral pigments.

### 3.2 Considering the utility of nanodiscs for creating spectrally-enhanced LHCs

The clear benefit of the nanodisc model membrane is the “confinement” effects limiting long-distance lipid diffusion.^84^ Here, the 2-D confinement of TR into a relatively small area close to LHCII has resulted in a structure where the majority of TR were likely to be within a few nanometres distance from an LHCII protein. The enhancement of LHCII fluorescence due to TR in nanodiscs is 262% relative to LHCII-only nanodiscs (in the 525–625 nm range); our previous study of TR–LHCII proteoliposomes revealed a maximal enhancement of LHCII with a similar TR : LHCII ratio of 120%.^60^ This difference is due to the significant fluorescence self-quenching effects which are known to occur in liposomes, attributed to protein–protein interactions between multiple LHCIIs.^47^ Our data clearly demonstrate the benefit of using nanodiscs to minimize protein–protein interactions and energy-dissipative effects towards achieving maximal enhancement of the fluorescence of a single LHC. Although these samples lacked the complications associated with LHC–LHC interactions, heterogeneous sub-populations were still present, including up to 35% of nanodiscs containing DOPC and TR but not LHCII (see footnote^85^ and ESI,† 2.10). However, spectroscopic measurements of LHCII photophysics, including LHCII emission and ultrafast TA, enabled characterization of rapid and efficient energy transfer from TR to LHCII.

While the nanodiscs provide an excellent platform for studying the interactions between lipid-tagged TR and single trimeric LHCII proteins, for this idealized system to be robust for industrial application, some practical aspects, *e.g.*, sample stability over time, may need to be evaluated and optimized. We have not assessed the stability of our nanodiscs beyond the measurement period of 1–3 days (see methodology, ESI,† Section 1.2), but we note that other studies have focused on the stabilisation of lipid bilayers containing proteins for long-term applications towards bio-hybrid devices.^86,87^

### 3.3 Comparison of non-covalent *versus* covalent strategies for interfacing chromophores and proteins

In this section, we briefly discuss the comparison between our non-covalent TR–LHCII system and systems using covalent attachments of synthetic pigments that have been reported before. Early studies demonstrated a transfer efficiency of >98% from single rhodamine red pigments covalently linked to trimeric LHCII proteins, resulting in an increase of >200% in the effective absorption strength at the protein's natural absorption minimum.^52^ In comparison, a study using LH2 complexes from purple bacteria covalently linked to Alexa647 pigments showed that the attachment of multiple pigments per protein (nine per LH2) resulted in a much greater effective increase in the absorption. With multiple energy transfer steps occurring at time constants between ∼0.5–20 ps leading to an overall transfer efficiency of ∼85%, this system exhibited a 12-fold increase of the absorption in the natural minimal spectral region.^54^ The absorption range of Reaction Centre (RC) protein complexes can also be enhanced by coupling them to non-native pigments, resulting in increases to the photochemical activity of isolated proteins^59,88^ and to the photocurrent generated by devices.^55^ In summary, our TR–LHCII system demonstrated absorption enhancement of ∼262% in the green spectral region and transfer times of 3–120 ps is comparable to these previous reports without the synthetic complexity of covalent attachment schemes. Therefore, the non-covalent strategy of introducing complementary chromophores into the lipid bilayer instead of directly cross-linking them to the protein is a viable method of artificially enhancing the absorption cross-section of an LHC. A distinct advantage offered by our self-assembly strategy is the modularity/flexibility for choosing both the pigment concentration and pigment type, without the concern for limiting factors such as a finite number of potential binding sites for attachment chemistry or genetic modification. Future studies could explore this modularity more broadly and expand the scope of this non-covalent strategy for constructing bio-hybrid light-harvesting systems using other proteins, pigments, and synthetic pigments. Our group recently developed “hybrid photosynthetic membranes” that are comprised of natural thylakoids merged with supported lipid bilayers into microarray patterns; these have the distinct advantage of being amenable to high-resolution microscopy.^89,90^ These membranes have the potential to be used for investigating energy transfer from non-covalent, lipid-tagged, spectrally complementary chromophores to LHC proteins in a highly complex photosynthetic system.

## 4 Conclusions

In this work, we established that interfacing LHCs to external chromophores *via* non-covalent self assembly can produce energy transfer on similar timescales to inter-protein transfer of excitation energy of the natural light-harvesting antennae.^78^ We have demonstrated that lipid nanodiscs are ideal for the study of isolated single membrane proteins as they can promote lipid–protein interactions and minimize any protein–protein interactions, which may distort the dynamics. In the future, other small organic chromophores could be incorporated into the lipid bilayer as an alternative to TR, such as molecules expected to have favourable chemical associations to the protein or those with favourable spectral characteristics or photophysical properties. For example, small hydrophobic molecules such as chlorins or Chl mimics,^91^ which will partition directly to the central hydrophobic portion of the lipid bilayer, may enable even faster energy transfer if they align more centrally to the Chls embedded within the LHC. Pigments that absorb at other wavelengths could be used to align to the “spectral gaps” of other LH proteins (*e.g.*, purple bacterial complexes).^54,55,59^ Finally, synthetic organic compounds developed for artificial photosynthesis, such as molecular dyads and triads consisting of energy/electron donor and acceptor moieties,^92,93^ could be added to the membrane and may couple to LHCs in a manner analogous to what we have demonstrated possible with external chromophores in this study. These model membrane systems have significance as idealised platforms to elucidate the photophysics and interactions between complementary chromophores and membrane proteins relevant to both natural and artificial light-harvesting systems.

## 5 Experimental section

A full experimental section describing the materials, methods and analysis is given in the ESI.† All relevant raw and analysed data associated with this paper are openly available under a CC-BY license in the Research Data Leeds repository^94^ and can be found at https://doi.org/10.5518/1020.

## Author contributions

A. M. H. and P. G. A. conceptualised the project. A. M. H. prepared all samples and performed initial characterisation. M. S. and M. N. performed TRFS and TA spectroscopy experiments and data analysis. T. W. performed MD simulations. C. D. P. D. performed energy transfer calculations. L. J. C. J. supported the development of biochemical procedures. P. G. A. and G. S. S.-C. supervised the overall project. A. M. H. and M. S. wrote the first draft of the manuscript. All authors contributed to revising the manuscript.

## Conflicts of interest

There are no conflicts of interest to declare.

## Supplementary Material

CP-023-D1CP01628H-s001
