# Supplementary material for: Ultrafast energy transfer between lipid-linked chromophores and plant light-harvesting complex II
Source: Phys Chem Chem Phys. 2021 Aug 26;23(35):19511–24. doi: 10.1039/d1cp01628h (PMC8442836; doi:10.1039/d1cp01628h)
Supplement: CP-023-D1CP01628H-s001 [file CP-023-D1CP01628H-s001.pdf]

Electronic Supplementary Material (ESI) for Physical Chemistry Chemical Physics.
This journal is © the Owner Societies 2021

Electronic Supplementary Information (ESI) for

# Ultrafast energy transfer between lipid-linked chromophores and plant Light-Harvesting Complex II

Ashley M. Hancock,[a,b,‡] Minjung Son,[c,d,‡] Muath Nairat,[c] Tiejun Wei,[e] Lars J. C. Jeuken,[b,f,g] Christopher D. P. Duffy,[f] Gabriela S. Schlau-Cohen[c,*] and Peter G. Adams[a,b,*]

[a] School of Physics and Astronomy, University of Leeds, Leeds LS2 9JT, UK.

[b] Astbury Centre for Structural Molecular Biology, University of Leeds, Leeds LS2 9JT,UK.

[c] Department of Chemistry, Massachusetts Institute of Technology, 77 Massachusetts Ave, Cambridge, MA 02139, USA.

[d] Department of Chemistry, University of Wisconsin−Madison, 1101University Ave, Madison, WI 53706, USA.

[e] School of Biological and Chemical Sciences, Queen Mary University of London, Mile End Road, London E1 4NS, UK.

[f] Faculty of Biological Sciences, University of Leeds, Leeds LS2 9JT, UK.

[g] Leiden Institute of Chemistry, Leiden University, 2300 RA Leiden, The Netherlands.

[‡] These authors contributed equally to this work.

[*] Correspondence: gssc@mit.edu; p.g.adams@leeds.ac.uk

## Table of Contents

1. Supplementary Materials and Methods
2. Supplementary Data
3. Supplementary References

# 1. Supplementary Materials and Methods

## 1.1. Materials.

All solvents and chemical solids were purchased from Sigma-Aldrich, UK, unless otherwise stated. Solvents were HPLC grade or higher and chemical solids BioUltra analytical grade or higher. All water used was deionized and passed through milli-Q water purification system before use. The fluorescently-tagged lipid known as Texas Red 1,2-dihexadecanoyl-sn-glycero-3-phosphoethanolamine (TR-DHPE) was purchased as a solid from Life Technologies (Invitrogen). Asolectin lipid extract from soybeans (Soy Asolectin) was purchased from Sigma-Aldrich in a dry granulated form. The detergent *n*-dodecyl α-D-maltoside (α-DDM) was purchased from Generon (Anagrade ≥99 % purity, high-alpha)

## 1.2. Sample preparation.

The major antenna protein of plants, trimeric LHCII, was extracted from spinach and biochemically purified as previously described (see **Fig. S1**) [1]. Soy Asolectin lipids were used as purchased: this lipid extract was used because of previous reports to give excellent LHCII stability for nanodiscs [2]. The TR chromophore tethered to a DHPE lipid was used as purchased (TR-DHPE), as is typical for lipid bilayer studies [3]. The ApoE422K belting protein which forms the scaffold for nanodiscs was produced in bacteria and purified as previously described [2]. Analysis of the belting protein by gel electrophoresis confirmed a high purity (see **Fig. S2**).

Lipid stocks were prepared for the formation of nanodiscs and liposomes by initially dissolving a known mass of Soy Asolectin lipids with a chloroform: methanol mixture (5:2). TR-DHPE was then dissolved into chloroform and added to the lipid stocks when required, always at a molar ratio of 75:1 Soy Asolectin: TR-DHPE (1.3 % mol/mol), this concentration aligns with a highest TR-to-lipid ratio previously used for TR-LHCII proteoliposomes [4], and was chosen because a relatively high concentration of TR is necessary to achieve comparable LHCII and TR absorbance which allows high signal-to-noise for ultrafast spectroscopy. Mixed lipids in organic solvents were then carefully dried to a thin film under clean nitrogen gas. These lipid thin films were then placed in a vacuum desiccator to remove any residual solvent (~3 hours). Lipid stocks were flash frozen in liquid nitrogen and stored at -80°C.

Liposomes (lipid vesicles) were prepared following standard procedures involving probe sonication [4]. Nanodiscs containing LHCII were prepared following a previously published protocol with minor modifications as described briefly below [2]. For nanodisc formation, lipid stocks were taken from the freezer, brought to room temperature, and then incubated in an detergent-containing aqueous solution overnight (20 mM HEPES, 100 mM NaCl, 0.5% α-DDM, pH 7.5), in order to solubilise lipids into detergent micelles. Nanodisc belting proteins (ApoE422K monomers), detergent-solubilised lipids and LHCII trimers were mixed in a 12.5: 3000: 1 ratio and incubated at 4°C for one hour with shaking. Polystyrene beads (Bio-Beads SM-2 adsorbents, Bio-Rad) were then added to samples up to 2/3 of the overall sample volume and incubated at 4°C with shaking for one hour to remove detergent. During this process the LHCII-lipid-TR nanodiscs will self-assemble. Nanodisc samples were then purified through a Ni-NTA affinity column to remove any material that was not incorporated into the histidine-tagged nanodiscs, a representative UV chromatogram from this stage is shown in **Fig. S3**. During Ni-NTA purification the running buffer for column binding contained 40 mM Imidazole, this was increased to 400 mM Imidazole for the column elution buffer. The sample was then dialysed, using 10,000 Da cutoff dialysis tubing, into a new buffer of 20 mM HEPES 100 mM NaCl (pH 7.5) to remove any residual elution buffer from the Ni-NTA column.

Immediately after formation, samples were stored at 4°C and only brought to room temperature for steady state spectroscopy measurements (typically for less than 1 hour) and were maintained at 4°C during longer TCSPC and TA measurements. Over the measurement period (1-3 days after sample formation), we note the following: (i) cold temperature storage (4°C) was always used (and is recommended), (ii) there were no peak shifts observed in the linear absorption spectra (i.e., the Chl a Qy absorption maximum remained stable at ~673 nm), (iii) LHCII and TR photobleaching is 1-5% for typical steady-state and TCSPC acquisitions of this kind (judged from a rapid steady-state fluorescence spectrum before vs after dataset acquisition). Long-term stability was not assessed in the current study and we note that for future work assessing any industrial applications it would be necessary to perform a systematic analysis of the stability.

**1.3. Steady-state absorption, fluorescence emission, fluorescence excitation spectroscopy.**
Before spectroscopy measurements, proteoliposome samples were diluted in a buffer of 40 mM NaCl, 20 mM HEPES (pH 7.5), to obtain a large enough volume for use in a 1 x 1 cm quartz cuvette (3 to 3.5 mL) at a low enough absorbance of ≤0.1 (per cm) at 675 nm to avoid inner filter effects [5]. Absorption spectroscopy was performed using an Agilent Technologies Cary 5000 UV-Vis-NIR absorption spectrophotometer equipped with an "integrating sphere" (also called a Diffuse Reflectance Accessory, Agilent) to remove any minor scattering effects.

Steady-state fluorescence spectroscopy was performed using an Edinburgh Instruments FLS980 fluorescence spectrophotometer equipped with dual excitation monochromators and dual emission monochromators. Samples were maintained at 20 °C and gently stirred during all measurements using a thermoelectrically cooled cuvette-holder with magnetic stirring capabilities (Quantum Northwest TC 1 Temperature Controller). For steady-state emission spectra, a 450W Xenon arc lamp was used for excitation and a red-sensitive-PMT for detection (Hamamatsu R928 PMT). Emission scans with selective excitation of LHCII were acquired with excitation at 473 nm collecting emission from 500-800 nm (2 nm and 1 nm bandwidth excitation and emission slits, respectively). Emission scans with selective excitation of Texas Red were acquired with excitation at 540 nm collecting emission from 550-800 nm (1 nm bandwidth for both excitation and emission slits). Data acquisition parameters were 0.5 nm steps, integrating 0.1 s/ step and five scans averaged for all. Fluorescence excitation measurements were acquired with emission collected at 686 nm (to be selective for LHCII emission) and scanning over an excitation range of 380 - 800 nm (1 nm and 2 nm bandwidth for excitation and emission slits, respectively). Data acquisition parameters were 1 nm steps, integrating 0.1 s/ step and a single scan for all samples.

**1.4. Time-resolved fluorescence spectroscopy.**
The fluorescence decay profiles were measured by time-correlated single photon counting (TCSPC). The excitation source was generated by coupling the output of a Ti:sapphire oscillator (MaiTai BB, SpectraPhysics; centered at 800 nm, 80 MHz repetition rate) into a nonlinear photonic crystal fiber (FemtoWhite 800, NKT Photonics), and then passing the generated white light through a 550 nm bandpass filter (ET550/15x, Chroma) to selectively excite the TR donor. The excitation beam was focused into a 100 $\mu$m spot on a 1 cm pathlength quartz cuvette containing the sample at an optical density of 0.08 per cm at 550 nm. The emission from the sample was detected by a single-photon-detecting avalanche photodiode (PDM Series, Micro Photon Devices), and the arrival time of each photon was recorded by a timing module (PicoHarp 300, PicoQuant, Inc.). Two different emission filters were used to selectively monitor the fluorescence of the TR donor (Em1: 560–600 nm; RPE560LP, Omega and FES0600, Thorlabs) and LHCII acceptor (Em2: >665 nm; BLP01-647R-25, Semrock),

respectively, as shown in **Fig. S8**. The polarization of the excitation beam was vertical, and the emitted fluorescence was detected at a magic angle (54.7 degrees) using a polarizer.

The data were measured in two different modes. All of the individual fluorescence decay traces presented in this work were measured by integrating the fluorescence signal over each emission wavelength range (Em1 or Em2) for a higher signal-to-noise ratio. Spectrally-resolved fluorescence data were obtained with spectral interferometry in the time domain (Gemini, NIREOS), and then Fourier transformed to yield the time-resolved fluorescence spectra (TRFS) as shown in Fig. 2 of the main text. For TRFS measurements, a 560 nm longpass emission filter (RPE560LP, Thorlabs) was used. The resolution of the emission wavelength axis was 0.7 nm. The interferometer was combined with the timing module described above to give fluorescence decay traces at each wavelength. Further details about the spectral interferometry setup and data analysis can be found elsewhere [6].

The excitation pulse energy was 30 fJ for all three samples. The experiment was repeated at 10 fJ and 100 fJ, and no power dependence was observed. The instrument response function (IRF) was determined by measuring scattered excitation light using a dilute solution of colloidal silica (Ludox, MilliporeSigma) [7], and has a width of 50 ps. Fluorescence decay traces were fitted with a mono- or bi-exponential function using iterative reconvolution with the IRF.

**1.5. Transient absorption (TA) spectroscopy.**

Details of the experimental setup can be found elsewhere [8]. The pump and probe pulses were produced by white light generation by focusing the output of a Ti:sapphire regenerative amplifier (Libra, Coherent; centered at 800 nm, 5 kHz repetition rate) into a chamber filled with pressurized argon gas at 20 psi. The residual fundamental and near-infrared region of the white light were filtered out with the combination of a dichroic mirror (DMSP805, Thorlabs) and a bandpass filter (FGS600, Thorlabs). For preferential excitation of the TR donor, the pump pulse was further passed through a bandpass filter (FES0600, Thorlabs), which resulted in the spectrum shown in **Fig. S10A**. With this pump spectrum, we estimate a 20.5% direct excitation of the higher vibronic bands of chlorophyll $Q_x/Q_y$ states in LHCII [9]. The spectrally filtered pump pulse was compressed with chirped mirrors (PC70, Ultrafast Innovations). The temporal width of the pump pulse was measured to be 26 fs by autocorrelation (**Fig. S10B**), and the dispersion in the probe was corrected for after data collection. The pump and probe pulses were focused into a 150 $\mu$m spot on a 1 mm pathlength quartz cuvette containing the sample at an optical density of 0.25/mm at the absorption peak maximum of each sample. The pump pulse was modulated with a chopper at 2.5 kHz, and the change in probe intensity was collected shot by shot at 5 kHz with a home-built spectrometer containing a linear CCD (Aviiva EM4-BA8, e2v Imaging). The excitation pulse energy was 6 nJ, which corresponds to $2.26\times10^{13}$ photons per pulse per $cm^2$, previously reported to be in the linear regime [10]. The transient absorption measurements were performed with vertical pump and vertical probe polarization. The sample was continuously flowed with a peristaltic pump to prevent photodegradation and repetitive excitation of the same spot. The sample reservoir was kept at 4°C throughout the measurement. Global analysis of the TA data was performed using the Glotaran software [11, 12].

**1.6. Size characterization of nanodiscs.**

Dynamic light scattering (DLS) measurements of liposomes and nanodiscs were performed using a ZetaSizer Nano (Malvern, UK). When acquiring data on a sample set, DLS was typically performed after absorption and fluorescence spectra had been acquired, in standard

sample buffers, using 80 $\mu$L polystyrene disposable cuvettes. Measurements were taken using a backscatter geometry (collection at 173°) and with a 633 nm laser probe. Temperature was set to 20 °C and at least three independent DLS runs were performed for each sample.

Transmission electron microscopy (TEM) was performed as follows. Nanodisc samples were prepared as described in 1.1 and diluted 10x in a buffer of 20 mM HEPES, 100 mM NaCl (pH 7.5). 5.0 $\mu$L of diluted nanodisc sample was deposited onto freshly glow-discharged carbon-coated copper grids and incubated for 30 s (grids were prepared in-house at the Astbury Centre TEM Facility at University of Leeds). Samples were then washed with 20 mM HEPES, 100 mM NaCl (pH 7.5) and then 5 $\mu$L of 1% uranyl acetate was added for a further 30 s incubation. Sample and buffer were then removed from the carbon grid which was then left at room temperate until completely "air dry". TEM was performed using a FEI T12 transmission electron microscope (FEI Tecnai T12, USA) at a range of magnifications. Analysis of particle sizes was performed manually using ImageJ.

## 2. Supplementary Data

### 2.1. Biochemical data on the purification of LHCII protein.

The major antenna protein of plants, trimeric LHCII, was extracted from spinach and biochemically purified as described in [1]. The LHCII protein's trimeric state and high degree of purity was confirmed by polyacrylamide gel electrophoresis (PAGE), as shown in **Fig. S1**.

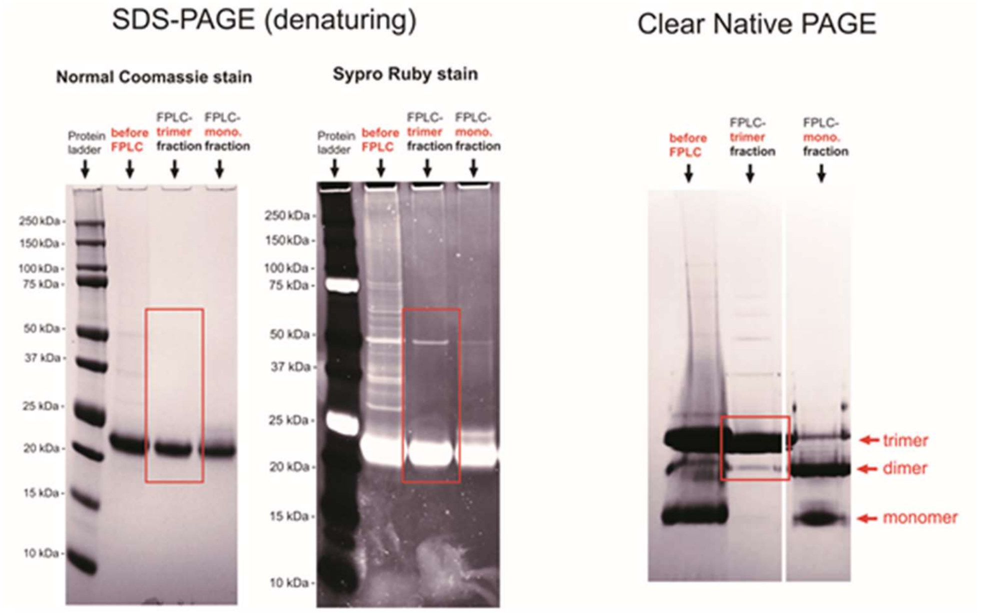


**Fig. S1.** PAGE to assess the purity of LHCII protein after its biochemical isolation. SDS PAGE gel with either Coomassie stain (*left panel*) or SYPRO-RUBY stain (*middle panel*). Gel lanes show, in order left to right: protein standard (with masses indicated), LHCII "before FPLC" (containing impurities, i.e., before size exclusion chromatography), purified trimeric LHCII (i.e., trimer fractions after size exclusion chromatography), purified monomeric LHCII (monomer fractions after size exclusion chromatography). Native-PAGE gel (*right panel*) was ran at 4°C and then stained with Coomassie. Native gel lanes show, in order: LHCII "before FPLC", purified trimeric LHCII, and purified monomeric LHCII, as above. Electrophoresis methodology was as previously described [1].

**2.2. Biochemical data on the purification of the ApoE422K belting protein.**

The ApoE422K belting protein was purified as described in methodology section 1.2 of this document. Its purity was confirmed by polyacrylamide gel electrophoresis (PAGE), as shown in **Fig. S2**.

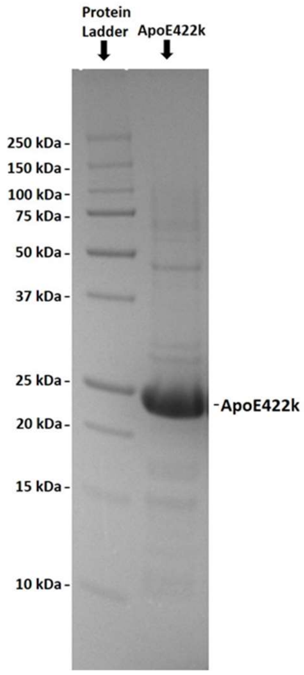


**Fig. S2.** PAGE to assess the purity of the nanodisc belting protein after its biochemical isolation. SDS-PAGE gel with Coomassie blue stain. Gel lanes shown are, left to right: protein standard ladder (with masses indicated) and the ApoE422K membrane scaffold protein (nanodisc belting protein). Image analysis using ImageJ software shows ~90% of signal in the ApoE422K lane is associated with the band situated at ~22kD, which is the expected size of the belting protein.

**2.3. Chromatography data from the nanodisc purification.**

The UV absorption trace of the Ni-NTA (nickel-nitrilotriacetic acid) column purification of LHCII nanodiscs, where the ApoE422k belting protein contains the histidine tag. **Fig. S3** is measured as liquid leaves the column and is collected into fractions. The initial peak (labelled *Load sample*) in the UV trace relates to the increased UV absorption of lipids and proteins which have not been reconstituted into the nanodiscs leaving the column. The flat section (labelled *Equilibrate*) relates to the washing step which ensures all unbound material has left the column. The arrow labelled *Elute* indicates the point where a high imidazole buffer is used to un-bind histidine-tagged nanodiscs from the column, and the sharp peak immediately after (labelled *Sample peak*) is caused by the increased UV absorption of nanodisc sample and is collected. The baseline is higher after elution than before elution due to the higher concentration imidazole buffer used for elution having increased UV absorption (this does not affect the sample collection as the sharp peak due to the sample can be easily resolved).

Successful formation of nanodiscs was also qualitatively assessed from the colour of eluted fractions after Ni-NTA purification. An observed pigmentation in eluted sample fractions of either green (for LHCII nanodisc samples) or grey (for TR-LHCII nanodisc samples) at the expected time during chromatography, i.e., only after adding the elution buffer, was a first suggestion that binding and then elution was successful and that the majority of LHCII-associated pigments and Texas Red were within nanodiscs.

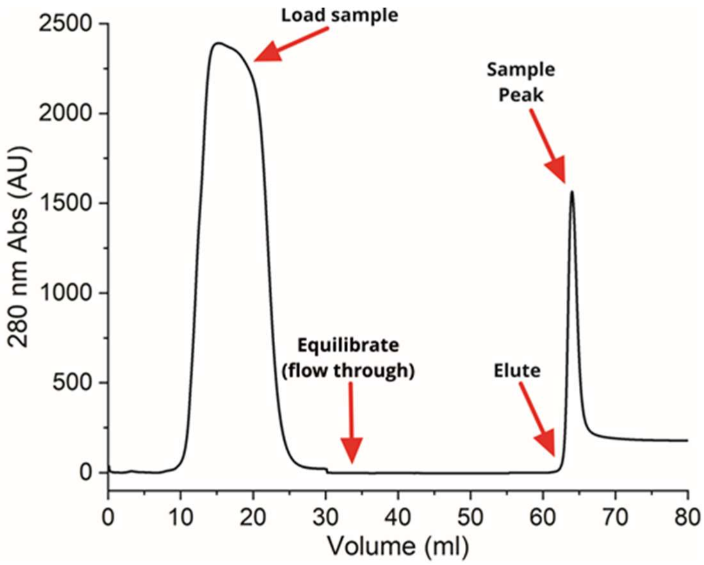


**Fig. S3** UV absorption trace showing the Ni-NTA column purification of LHCII nanodiscs.

**2.4. Size characterization of nanodiscs.**

Transmission electron microscopy (TEM) images of the representative LHCII-only nanodisc sample at different levels of magnification are shown in **Fig. S4A-D**. A histogram of the nanodisc diameters that were measured (n=107) is shown in **Fig. S4E**. The TEM images show a distribution of nanodisc diameters with an average of 21±7 nm (error value is the calculated standard deviation). This value is slightly lower than the expected size of LHCII loaded nanodiscs of 25-30 nm [13]. However, this is not surprising because "unloaded" nanodiscs were also anticipated due to the deliberate excess of ApoE422K and lipids that were used (necessary to bias assembly towards 1 LHCII per disc) and it is known that these lipid-only nanodiscs typically have a smaller diameter [14]. The size distributions for nanodiscs measured with dynamic light scattering (DLS) are shown in **Fig. S4F-G**: LHCII nanodiscs (*green line*), TR-LHCII nanodiscs (*blue line*) and TR liposomes (*red line*) are displayed. The DLS graph for "intensity vs size" shows a consistent difference in size for the populations of liposomes vs the population of nanodiscs. For each sample, three repeat measurements were taken and importantly a single roughly Gaussian distribution was found for all samples which suggested a mostly homogeneous population, rather than multiple peaks which would be found for heterogeneous samples (see **Fig. S4F**). The absolute values for size are not accurate, because DLS tends to overestimate the size of any distribution because larger particles scatter much more than smaller particles. Conversion of this data to "volume vs size" and averaging give a more accurate representation of the size (see **Fig. S4G**). The positions of the peak maxima of the volume/size distributions can then be assessed. Overall, this leads to a best estimate of 24 nm, 28 nm and 42 nm for the LHCII nanodiscs, TR-LHCII nanodiscs and TR liposomes, in good agreement with the TEM data for the representative nanodisc sample.

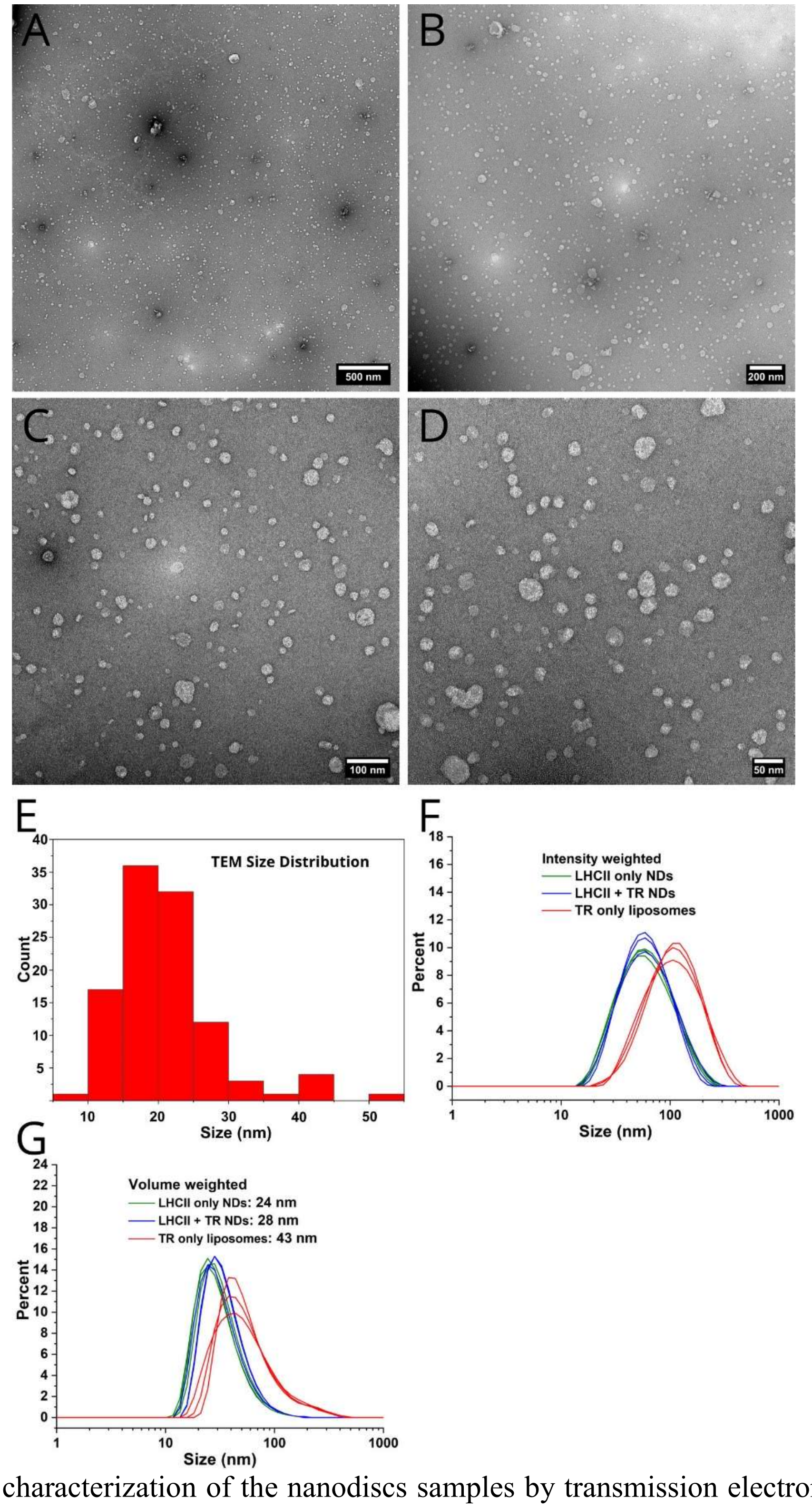


**Fig. S4.** Size characterization of the nanodiscs samples by transmission electron microscopy (TEM) and dynamic light scattering (DLS). (A)-(D) TEM images of LHCII only nanodiscs at different levels of magnification. (E) Size distribution histogram of nanodisc diameter in TEM images (n=107), images were analyzed using the ImageJ software's 'measure' function and manually drawing profiles across the centers of each particle in the images. (F-G) DLS particle distribution data comparing the nanodisc and liposome samples (n=3 for each sample). (F) Intensity-weighted size distribution. (G) Volume-weighted size distribution, where data is weighted by the volume of the particle. In DLS, intensity^3 is proportional to volume.

### 2.5. Comparison of the absorbance spectrum of LHCII between different samples as prepared: in detergent, proteoliposomes and nanodiscs.

The only noticeable absorption difference in the LHCII absorption spectra is a slight blueshift of the peak representing the Chl *a* $Q_y$, from 675 to 674 nm from detergent to nanodiscs, which could reflect the change in local environment from detergents to lipids. Similar subtle spectral shifts for LHCII in membrane discs as compared to detergent have previously been reported [2]. This compares favourably to typical LHCII proteoliposome samples where more significant changes in spectral shape and blue-shifts of 1-3 nm are typical [3, 15].

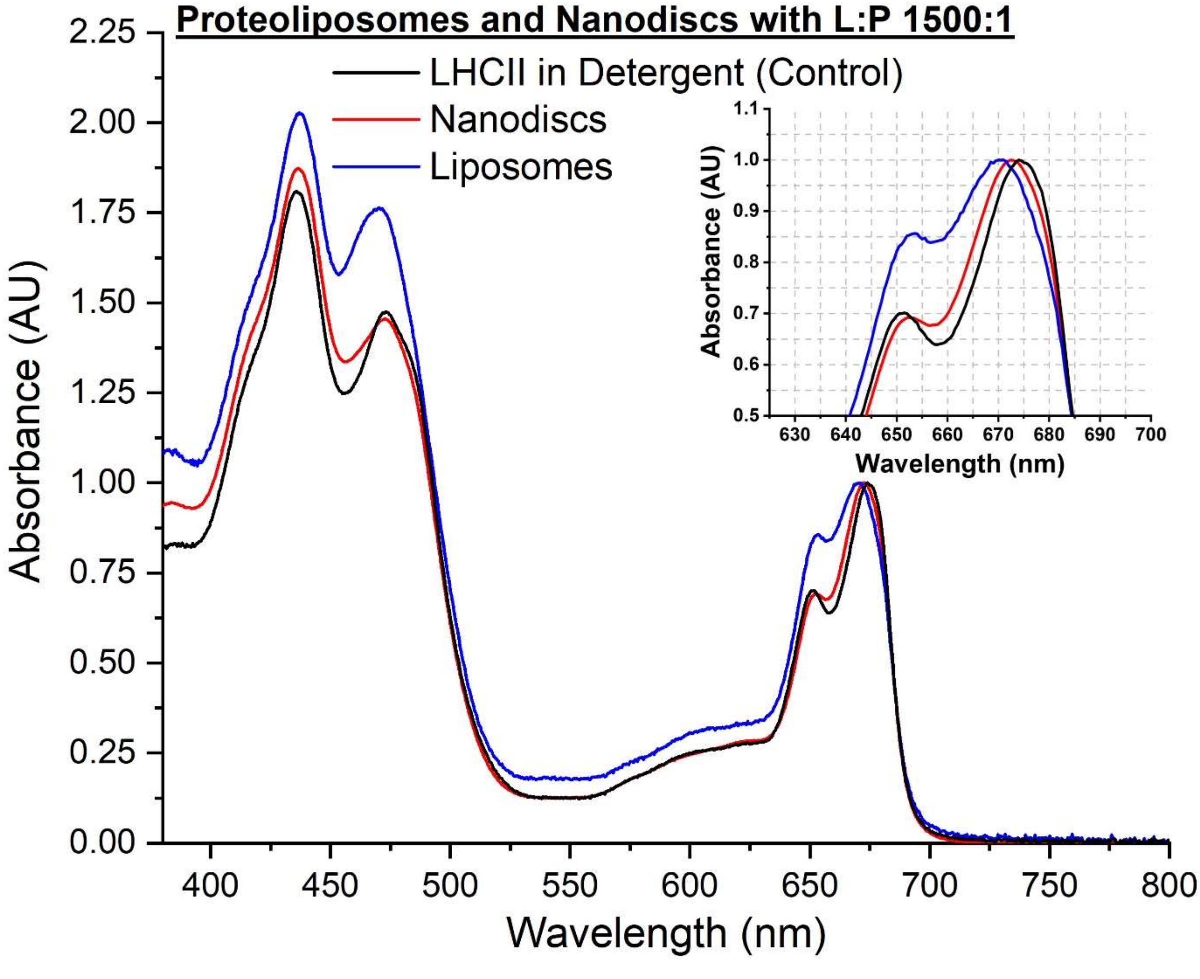


**Fig. S5.** Comparison of absorption spectra of: (i) LHCII reconstituted into liposomes (i.e., proteoliposomes), (ii) LHCII reconstituted into nanodiscs and (iii) isolated LHCII (in detergent micelles, as purified). Small spectral shifts are observable for LHCII within liposomes and nanodiscs relative to the starting material of isolated LHCII. All spectra are normalised to 1.0 at the Chl *a* absorption peak at ~675 nm for visual clarity.

## 2.6. Decomposition of absorption and emission spectra in order to separate the contributions due to LHCII and TR.

As part of the process of quantifying the concentration of Texas Red, the absorption spectrum of the TR-LHCII nanodisc was decomposed into components originating from the absorption of TR and LHCII, because LHCII overlaps throughout the TR absorption range. To obtain the "TR-only" component, a representative LHCII absorption spectrum, collected in detergent and normalized to the Chl *a* $Q_y$ peak at 675 nm, was subtracted from the TR-LHCII spectrum, to give results as shown in **Fig. S6A**.

To quantify the relative fluorescence intensity of samples (see section 2.8), the LHCII and TR component peaks within fluorescence emission spectra were also decomposed in a similar manner to absorption spectra. To obtain the "LHCII-only" component of fluorescence, a representative TR emission spectrum, collected from TR within liposomes, was normalised to the TR emission peak at 610 nm and then subtracted from the TR-LHCII spectrum (**Fig. S6B**). To obtain the "TR-only" fluorescence component, a representative LHCII emission spectrum was normalised to roughly 80% of the TR-LHCII emission at ~681 nm (LHCII Chl *a* $Q_y$ peak) and then subtracted, resulting in spectra as in **Fig. S6C**. This % value was found empirically (via multiple iterations) to be the optimal value which produces a decomposed spectrum for TR: this is because the value for TR emission at 681nm is known to be roughly 20% of its peak maximum at 610 nm (as determined from fluorescence spectroscopy of pure isolated Texas Red). The end result of the decomposition procedure for the TR-LHCII nanodisc fluorescence spectrum is shown in main text Fig. 1C (*solid lines*).

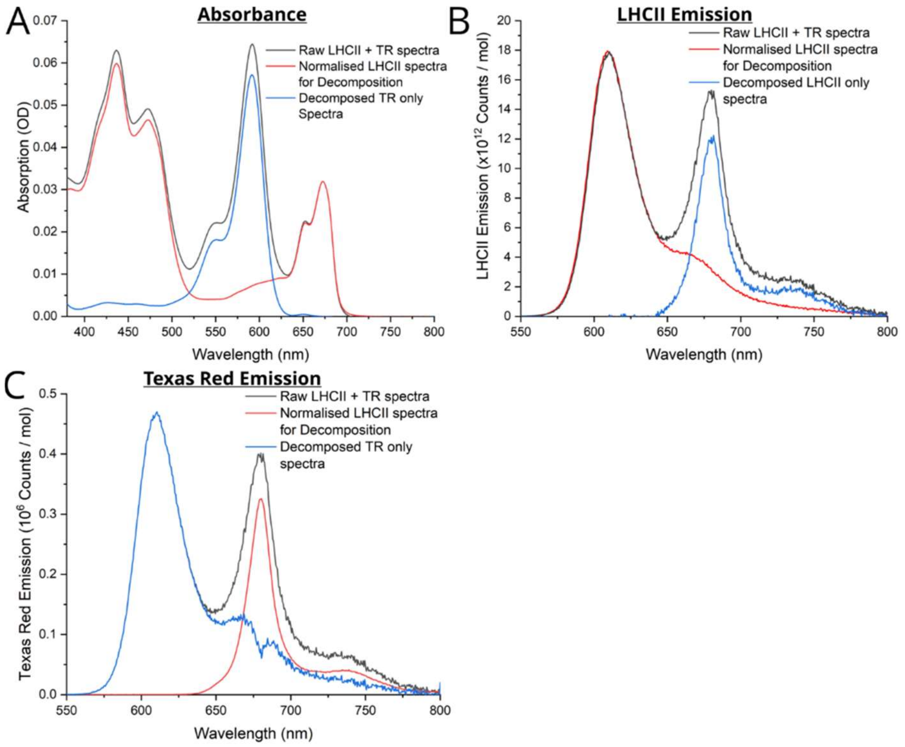


**Fig. S6.** Example results of the manual spectral decomposition procedure described. Analysis is shown for isolating: (A) TR absorbance, (B) LHCII fluorescence emission, and (C) TR fluorescence emission. All analysis was performed using Origin Pro (v.9) graphing software. All absorption spectra were "baselined" to give an absorbance of zero at 800 nm as expected in these samples, to remove any slight differences in the background noise (any minimal background noise in fluorescence spectra is removed during data acquisition). All spectra were corrected for the factor by which each sample was diluted, by multiplying by the dilution factor.

**2.7. Quantification of the concentration of LHCII and TR after sample preparation.**
One cannot assume that samples at the end of a multi-step preparation procedure will contain the same amount of material as input into at the start of the procedure because many biochemical procedures are inefficient and some material may be lost. In order to quantify the final composition of our nanodisc and liposome samples, as prepared, we applied the well-known relationship that the concentration of an optically-active component is directly proportional to its absorbance (the Beer-Lambert law), where the proportionality constant is taken from the absorbance of sample at known concentration.

Thus, **LHCII content** was estimated from the absorbance in the region expected for the Chl *a* $Q_y$ peak of LHCII, specifically, from the integrated area under the absorbance spectrum from 635-700 nm and a conversion factor (absorption coefficient) to give a concentration in moles/liter. No spectral de-composition was needed, even for the TR-LHCII nanodisc sample, because there is no significant overlap of absorption from Texas Red >630 nm. Area was used to ensure that any slight peak broadening did not lead to a loss of relative absorption. Concentration was calculated as:

$$LHCII = \frac{1}{6.73 \times 10^6} \times \int_{635\,nm}^{700\,nm} A(\lambda)\, d\lambda$$

where the constant $6.73 \times 10^6$ $M^{-1}$ $cm^{-1}$ is an area-based molar absorption coefficient, i.e., the integrated area which represents 1 mol/L LHCII in a 1-cm pathlength cuvette as measured from a control sample of low concentration LHCII in 0.03 % α-DDM, 20 mM HEPES, pH 7.5.

Similarly, **Texas Red content** was assessed from the absorbance peak height at 591 nm and a conversion factor (absorption coefficient) with TR-DHPE concentration in moles/liter calculated. Here, peak height was used (as opposed to integrated area) because TR was never observed to undergo any spectral broadening or shifts. For the TR-LHCII nanodisc sample, a spectrally decomposed absorbance spectrum was used, as in **Fig. S6A** (for the TR liposomes sample this was not necessary as TR was the only optically-active component). Concentration was calculated as:

$$TR = \frac{peak\ height}{85{,}000}$$

where the constant 85,000 $M^{-1}$ $cm^{-1}$ is the molar absorption coefficient, i.e., the absorbance which represents 1 mol/L TR in a 1-cm path length. This value was provided by the supplier of TR-DHPE and is in agreement with other published works [16].

The **Total lipids** (Soy Asolectin) in the starting material was 1.523 mM for the nanodiscs samples and 4.193 mM for the liposomes sample.

**Table S1** shows the calculated values for LHCII and TR concentration for all samples assessed in the current study (*highlighted in red*). The molar ratios of lipid, TR and LHCII relative to each other can be calculated, in order to assess the relative effectiveness of their incorporation into model membranes, shown in **Table S2**. Specifically: (i) the TR–to-LHCII ratio was calculated simply by using the concentrations of TR and LHCII estimated from absorption spectroscopy, (ii) the LHCII-to-lipid ratio was calculated from the concentration of LHCII and assumed that there was no loss of lipid, and (iii) the ratio of TR-to-lipid was assumed to be maintained at 1:75, in other words, that the Soy Asolectin lipid had the same incorporation yield as the TR-tagged lipids, as is typical [17].

The TR:LHCII ratio achieved in the nanodisc sample was very close to the 40:1 ratio in the starting mixture. The LHCII nanodisc sample had an estimated lipid:LHCII ratio of ~3000:1. The TR liposome sample was prepared with the same lipid mixture as the TR-LHCII nanodiscs resulting in a 75:1 lipid:TR ratio. These values correspond to consistent yields of both LHCII and TR components with final ratios similar to the that in the starting material.

**Table S1.** Analysis of steady-state spectroscopy data from each sample in order to calculate: (i) concentration of LHCII, (ii) concentration of TR, (ii) energy transfer efficiency (ETE) and (iii) LHCII fluorescence enhancement. For both fluorescence emission measurements were made using excitation light that was selective for TR, to test the TR-to-LHCII transfer (excitation at 540 nm). The procedure for calculating the relative fluorescence, ETE and LHCII enhancement is detailed in the next section, **supplementary section 2.8**.

| **Sample description** | **Total lipids** | **LHCII Absorbance** | **LHCII Content** | **TR Absorbance** | **TR Content** | **Relative TR Fluorescence** | **ETE (steady -state)** | **Relative LHCII Fluorescence** | **LHCII Fluorescence Enhancement** |
|---|---|---|---|---|---|---|---|---|---|
| | (mM) | (integrated area 635-800 nm, dil. corrected) | (μM) | (peak height at 591 nm, dil. corrected) | (μM) | (peak height at 610 nm) | (%, relative to isolated TR) | (integrated area 625-800 nm) | (%, relative to isolated LHCII) |
| **LHCII nanodiscs** | 1.52 | 40.7 | 0.60 | - | - | - | - | $1.52 \times 10^{14}$ | 96.8 |
| **TR liposomes** | 1.52 | - | - | 4.73 | 55.9 | $1.23 \times 10^{12}$ | - | - | - |
| **TR-LHCII nanodiscs** | 4.19 | 35.9 | 0.53 | 1.72 | 20.3 | $4.68 \times 10^{11}$ | 62.0 | $4.00 \times 10^{14}$ | 255 |

**Table S2.** Calculated ratios of lipids to LHCII and/or TR components in all samples.

| **Sample description** | **Total lipids** | **Calculated concentration** | | **Lipid: LHCII** | **Lipid: TR** | **TR: LHCII** |
|---|---|---|---|---|---|---|
| | (mM) | **LHCII content** (μM) | **TR content** (μM) | (ratio) | (ratio) | (ratio) |
| **LHCII nanodiscs** | 1.52 | 0.60 | 0.0 | 2540 | - | - |
| **TR liposomes** | 4.19 | 0.00 | 55.9 | - | 75 | - |
| **TR-LHCII nanodiscs** | 1.52 | 0.54 | 20.3 | 2820 | 75 | 38 |

**2.8. Quantification of TR-to-LHCII energy transfer efficiency and quantification of LHCII fluorescence enhancement for TR-LHCII nanodiscs.**

Considering *either* the TR molecule *or* the LHCII protein, the "relative fluorescence intensity" was defined as the measured fluorescence intensity at the wavelengths of interest divided by its concentration (using the concentration calculated from absorbance data, as in section 2.7, and absolute values of fluorescence emission calculated from TR and LHCII emission spectra, as in section 2.6):

$$\boldsymbol{relative\ TR\ fluorescence} = \frac{measured\ TR\ emission\ counts}{calculated\ TR\ concentration}$$

$$\boldsymbol{relative\ LHCII\ fluorescence} = \frac{measured\ LHCII\ emission\ counts}{calculated\ LHCII\ concentration}$$

Then, the efficiency of resonance energy transfer can be calculated from the quenching of the fluorescence of the donor molecule, in our case, TR. Assessing this from the steady-state fluorescence spectroscopy data, **Energy Transfer Efficiency** is calculated by the following well-established relationship [18, 19]:

$$ETE = 1 - \frac{F_{DA}(TR)}{F_D(TR)}$$

where $F_{DA}$ and $F_D$ are the relative TR fluorescence in the presence or absence of LHCII, respectively. Here, $F_D$ represents fluorescence measured for TR within liposomes (i.e., the sample shown in the table) as the appropriate control sample where there are no TR-LHCII interactions and no quenching.

Next, the "acceptor enhancement" due to the presence of the energy donor molecule can be found. In our case, this is the increase in the fluorescence intensity from LHCII due to the energy transfer from TR. Assessing this from the steady-state fluorescence spectroscopy data, **LHCII fluorescence enhancement** is calculated as follows [4]:

$$LHCII\ enhancement = \frac{F_{DA}(LHCII)}{F_A(LHCII)}$$

where $F_{DA}$ and $F_A$ are the relative LHCII fluorescence in the presence or absence of TR, respectively. Here, $F_A$ represents fluorescence measured for LHCII isolated in detergent as the appropriate control sample where there are no protein-protein interactions and no quenching (measured as $1.57 \times 10^{14}$ counts).

**2.9. Energy transfer efficiency calculations using linear absorption and fluorescence excitation spectra.**

Comparison between fluorescence excitation spectra and linear absorption ("*1–Transmission*") spectra can indicate the connectivity of chromophores within LHCII, and we would expect perfectly overlapping spectra if all chromophores are well connected as all energy absorbed by the system would manifest as fluorescence from the terminal emissive state. Comparison of fluorescence excitation and absorption spectra can also be used to determine the energy transfer efficiency between donor and acceptor molecules which undergo FRET. These two types of spectra were collected for LHCII nanodisc samples with and without TR-DHPE. Fluorescence excitation spectra were collected by selectively collecting LHCII emission at 686 nm and scanning over a range of excitation wavelengths. Linear absorption spectra were calculated as one minus the sample transmission (*1 – T*). Sample transmission, *T*, is defined as the fraction of the incident beam intensity which is transmitted through the sample ($\mathrm{I}_T/\mathrm{I}_0$), can be obtained from a sample's absorbance using the relationship:

$$T = \frac{\mathrm{I}_T}{\mathrm{I}_0} = 10^{-Abs}$$

where the logarithmic absorption, *Abs*, is the standard measurement made by the instrument:

$$Abs = \log_{10}\left(\frac{\mathrm{I}_0}{\mathrm{I}_T}\right)$$

The excitation spectrum and linear absorption spectrum for LHCII nanodiscs (*green lines* in **Fig. S7**) are very closely matching suggesting that energy absorbed at any wavelength is transferred to the same terminal fluorescence emitter. This is excellent evidence that LHCII is intact and that the inter-pigment energy transfer pathway has not been disrupted when reconstituted into nanodiscs.

If the excitation spectrum shows lower intensity than the linear absorption there is less than 100% energy transfer between excitation donors and acceptors at that wavelength. The ratio of fluorescence excitation to linear absorption over the donor region is therefore directly related to ETE, which can be calculated as follows:

$$\boldsymbol{ETE = 1 - \frac{(1 - T_D) - Ex_D}{(1 - T_D)}}$$

where $T_D$ = Donor transmission maximum; $Ex_D$ = Donor excitation maximum.

For the TR-LHCII nanodiscs sample (*blue lines* in **Fig. S7**), the excitation spectrum appears to be around two-thirds of the height of the linear absorption spectrum over the region of TR (525-625 nm). Using the above equation, an energy transfer efficiency of 65% is calculated.

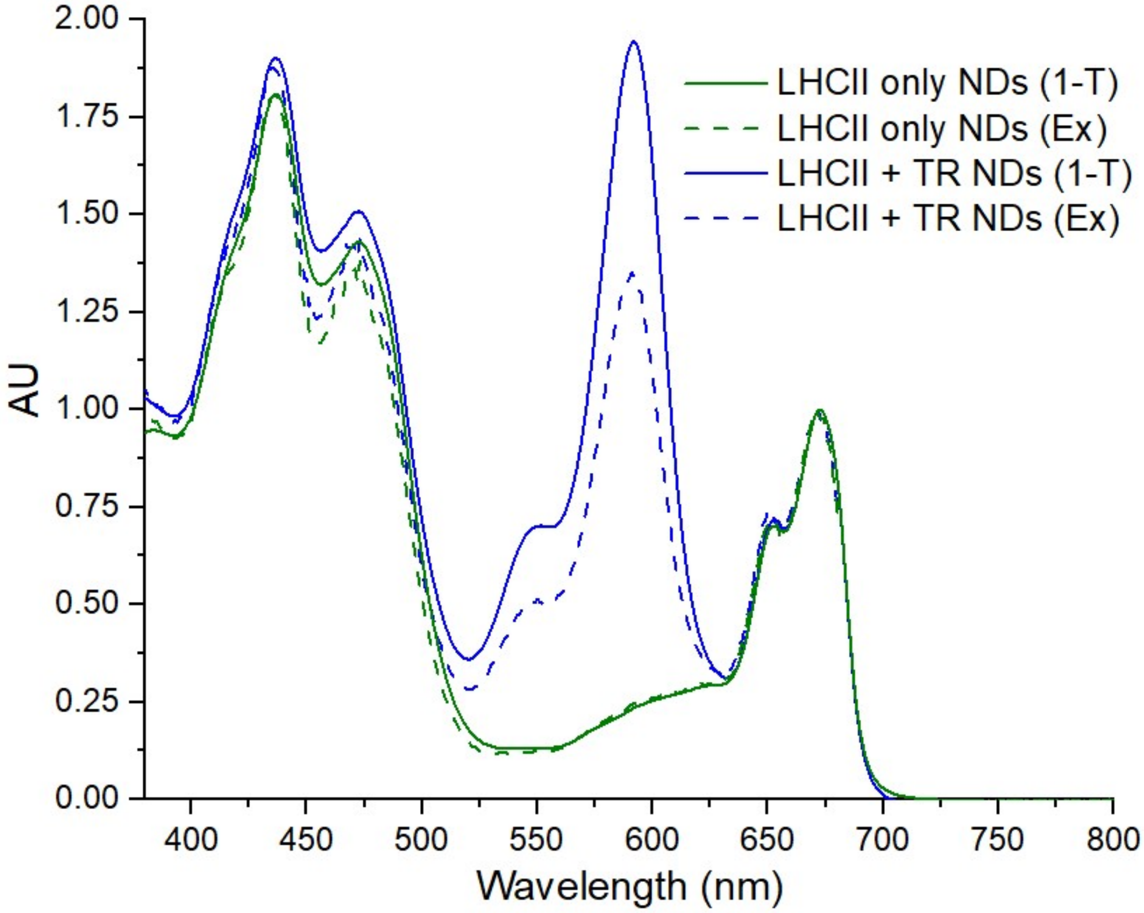


**Fig. S7.** LHCII fluorescence excitation (*Ex*) and linear absorption (*1-T*) spectra for LHCII nanodiscs with and without Texas Red. Fluorescence excitation spectra were collected monitoring the emission at 686 nm (selective for LHCII). Both spectra were normalised to 1.0 at the LHCII Chl *a* $Q_y$ absorption peak at ~675 nm, in order to allow comparison.

## 2.10 Estimating the proportion of TR only nanodiscs and true ETE in TR-LHCII nanodiscs

If we assume that nanodiscs have a composition of either 1500:20:1 DOPC:TR:LHCII (single LHCII nanodisc) or 1500:20 DOPC:TR (no LHCII) and our calculated ratio is 2870:38.3:1 then this implies that we would expect <50% of nanodiscs to be without LHCII. For these, the TR fluorescence intensity will be at its maximal "unquenched" level (i.e., donor-only).

**Calculation 1 – Estimating the fraction of nanodiscs that contain LHCII from steady-state fluorescence data of TR quenching and the time constants from TA:**

In our measurements, we will observe the quenched fluorescence of the ensemble population of nanodiscs, so if we term the two nanodisc sub-populations *ND-LHCII* and *ND-0* for those *containing LHCII* and those that *do not contain LHCII*, respectively:

$$Observed\ Fluor.Intensity, \qquad F(quenched) = \frac{F(TR_{ND-0}) + F(TR_{ND-LHCII})}{F(TR_{donor-only})}$$

*F(quenched)* was measured as 38% as calculated from Fig. 1C.

The fluorescence of each sub-population will be related to its fluorescence quantum yield *(QY)* and the proportion of the total population that it represents (*N* or *%*), as:

$$F = QY \cdot N$$

If we define the relative fluorescence quantum yield of each subpopulation as follows:

$$QY(TR_{ND-0}) = (TR_{donor-only}) = 1$$

and the relative percentages of $TR_{ND-0}$ and ($TR_{ND-LHCII}$) as $\boldsymbol{x}$ and ($\boldsymbol{100 - x}$).

So: $$F(quenched) = \frac{x + (100 - x) \cdot QY(TR_{ND-LHCII})}{100} \qquad \text{Eqn (1)}$$

We can calculate the relative fluorescence quantum yield expected for nanodiscs containing LHCII using the energy transfer time found from the TA data. If we consider only the 128 ps TA component (loosely-coupled TR):

$$Relative\ QY = \frac{original\ rate\ of\ decay}{original\ rate\ of\ decay + rate\ of\ additional\ decay}$$

$$QY(TR_{ND-LHCII}) = \frac{(\tau_{fl})^{-1}}{(\tau_{fl})^{-1} + (\tau_{ET})^{-1}} = \frac{(3940ps)^{-1}}{(3940ps)^{-1} + (128ps)^{-1}} = 0.0314$$

If we consider only the 3.7 ps TA component (tightly-coupled TR) then the calculation gives *QY*= 0.000938. Furthermore, within the nanodiscs containing TR and LHCII there may be a population of TR molecules that are not directly coupled to LHCII, however, these may transfer

excitation via TR-to-TR transfer to LHCII. These calculations are only estimates because we do not know the fraction of TR which is loosely- or tightly- coupled to LHCII from the global analysis, therefore it seems reasonable to use the slowest transfer to represent the minimum amount of quenching expected.

Substituting F(quenched) = 0.38 and QY($TR_{ND\text{-}LHCII}$) = 0.0314 into Eqn (1)

$$0.38 = \frac{x + (100 - x) \cdot 0.0314}{100}$$

$$38 = x + 3.14 - 0.0314x$$

$$34.86 = 0.9686x$$

$$x = \frac{34.86}{0.9686} = 36$$

In summary, we find that approximately 64% of nanodiscs contain both LHCII and TR and 36% of nanodiscs contain TR without LHCII.

**Calculation 2 – Estimating the fraction of nanodiscs that contain LHCII from time-resolved fluorescence data of TR quenching:**

We can also judge the fraction of nanodiscs with and without LHCII from the relative amplitudes of the fit for the TR fluorescence decay curve (TCSPC), if we assume that the fast component represents the nanodiscs containing both LHCII and TR and the slow component represents the nanodiscs where LHCII is absent (main text Fig. 2F and Table 1). From Table 1 we can see that the amplitude of the slow-decaying component ($A_2$) for the TR-LHCII (Em1) is 41.2%, in good agreement with the estimate from steady-state data. In other words, the TCSPC data suggests that approximately 59% of nanodiscs contain both LHCII and TR and 41% of nanodiscs contain TR without LHCII.

**Calculation 3 – Estimating the energy transfer efficiency for a hypothetical population of (pure) LHCII-TR nanodiscs:**

Considering only the fast component of TR decay ($\tau_1$ of 0.32 ns with $A_1$ = 58.8%, see main text Table 1), which we interpret as representing the TR lifetime in LHCII-TR nanodiscs and 58.5 % of the population, we can estimate the TR-to-LHCII transfer efficiency for this sub-population as:

$$ETE = 1 - \frac{\tau_1}{\tau_0}$$

where $\tau_0$ is the lifetime of isolated TR = 3.94 ns and $\tau_1$ = 0.32 ns.

Thus, the ETE for a population of pure TR-LHCII-nanodiscs is estimated as 91.9%.

### 2.11 Considering inter-TR energy transfer in nanodiscs

**Estimation of nanodisc size:**

If we assume that nanodiscs have a composition of 1500:20:1 DOPC:TR:LHCII (single LHCII nanodisc), we have 1520 lipid molecules in each lipid bilayer in a single nanodisc, therefore 760 lipids in each leaflet of the bilayer (including an average of **10 TR** in each leaflet).

If area/lipid = 0.66 nm$^2$ [20]

Total lipid area: 760 x 0.66 = **502 nm$^2$**

Area/ LHCII = 52 nm$^2$ [21]

Total occupied area = 554 nm$^2$

Leads to:

$A = \pi r^2$, $r = \sqrt{\frac{A}{\pi}}$, r = 13.3 nm$^2$ (therefore, nanodisc diameter = 26.6 nm$^2$)

This estimate generated from the expected molecular ratios and dimensions is in good agreement with the expected diameter of ~25-30 nm [13] and our measurements of ~21-28 nm from electron microscopy and dynamic light scattering data presented in ESI 2.4.

**Estimation of TR-TR separation in lipid nanodiscs**

Using the values estimated above for membrane area as 502 nm$^2$ and 10 TR per leaflet:

Concentration of TR (in each leaflet) = 10/502 nm$^2$ = 0.0199 TR/ nm$^2$ (or ~2 per 100 nm$^2$)

Therefore, area occupied per TR = 1/C = 50.2 nm$^2$

Therefore, average TR-TR molecular separation = $r = \sqrt{\frac{A}{\pi}}$ = **4.0 nm$^2$**

To summarize: from the ratio of labelled and normal lipids that we used, 1:75 TR-to-DOPC, we would expect a distribution of TR-TR centre-to-centre separations with a mean of ~4.0 nm.

**Estimation of TR-TR energy transfer efficiency in nanodiscs**

Using the conventional relationship between Förster radius ($R_0$) and inter-pigment distance (R), we can estimate the energy transfer efficiency [18]: $ETE = \frac{R_0^6}{R_0^6+R^6}$

$R_0$ was calculated to be 6.05 nm from the normalised absorption and fluorescence emission spectra of Texas Red using the online tool [22, 23] and the TR-TR separation distance of 4.0 nm was used (as estimated above).

Thus, we estimate energy transfer efficiency to be ~92% based on inter-TR separation of 4.0 nm and an $R_0$ of 6.05 nm:

$$ETE = \frac{R_0^6}{R_0^6 + R^6} \times 100\% = 92.2\%$$

### 2.12 Enhancement of overall system's absorption due to the addition of TR

In order to calculate the effective enhancement of the system's absorption due to the addition of Texas Red, the fluorescence excitation spectrum of LHCII nanodiscs and TR-LHCII nanodiscs were compared. The excitation spectra were used as they represent the "effective" absorption of the system, i.e., where the excitation energy is successfully transferred to the LHCII complex (leading to the fluorescence observed), as opposed to the simple steady-state absorption spectrum which would include "ineffective" TR molecules that were not actually coupled energetically to LHCII. Spectra were normalized to 1.0 at the Chl *a* $Q_y$ peak to allow comparison relative to the starting amount of LHCII in different samples.

As shown below, spectra were plotted in Origin Pro graphing software and the integration function was used to quantify the area under the spectrum in the specified range in each case:

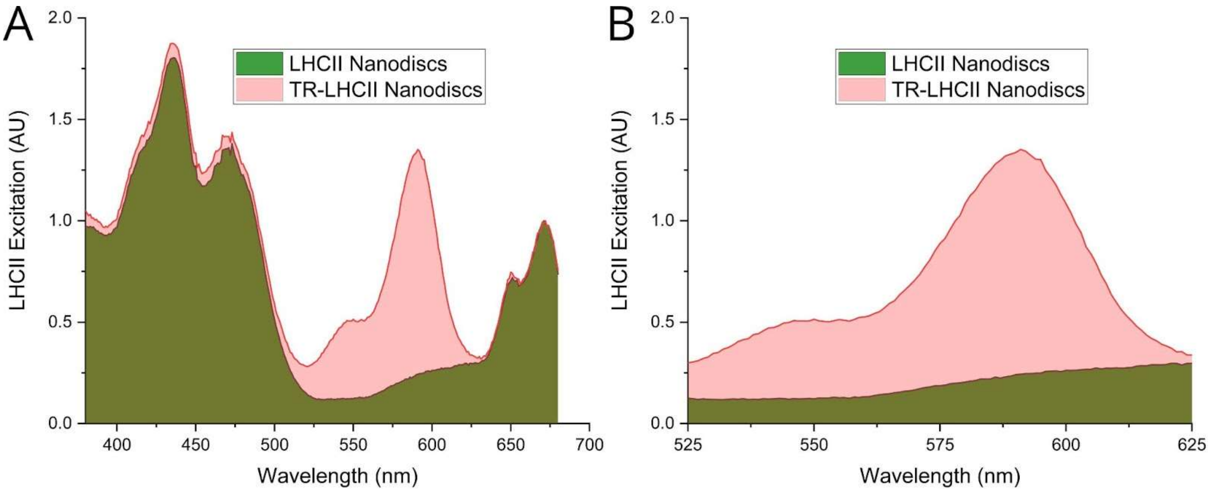


**Fig S8. Enhancement of LHCII excitation spectra** (A) LHCII excitation spectra (emission collected at 680 nm) normalized to 1.0 at Chl *a* $Q_y$ peak for LHCII nanodisc and TR-LHCII nanodisc samples. (B) zoomed in the region of (A) showing the "green gap" of minimal natural LHCII absorption between 525-625 nm.

**Full range:**

LHCII nanodiscs effective absorption area in the visible range 380-680nm (*green area* in ESI Fig S8A) = 208.4 AU

TR-LHCII nanodiscs effective absorption area in the visible range 380-680nm (*red are*a in ESI Fig S8A) = 270.3 AU

Absorption strength (enhanced vs. original) = 270.3/208.4 = 1.297

The enhancement of LHCII in nanodiscs over the entire visible range is estimated as 29.7% due to the presence of TR.

**Green gap:**

LHCII nanodiscs effective absorption area in the "green gap" 525-625nm (*green area* in ESI Fig S8B) = 19.4 AU

TR-LHCII nanodiscs effective absorption area in the "green gap" 525-625nm (*red area* in ESI Fig S8B) = 70.2 AU

Absorption strength (enhanced vs. original) = 70.2/19.4 = 3.62

The enhancement of LHCII in nanodiscs focussing on the "green gap" spectral range is 262% due to the presence of TR.

## 2.13 Enhancement of overall system's absorption due to the addition of TR considering the AM1.5 solar spectrum

In order to calculate the effective enhancement of the LH system's absorption due to the addition of Texas Red, the fluorescence excitation spectrum of LHCII nanodiscs and TR-LHCII nanodiscs was analysed (Fig. S9(A)), similarly to in ESI 2.12. Furthermore, to consider the system's absorption in relation to a real-life environment we normalised this excitation spectrum to the standard AM1.5 solar spectrum (Fig. S9(C)), available online from the global standards organization ASTM International [34]. In other words, the fluorescence excitation spectra were multiplied by the AM1.5 solar spectrum (Fig. S9(B)). The effective absorption area of these normalised spectra was then compared (with TR versus without TR). Full details are given below.

**Full range:**

As shown below, spectra were plotted in Origin Pro graphing software and the integration function was used to quantify the area under the spectrum in the specified range in each case:

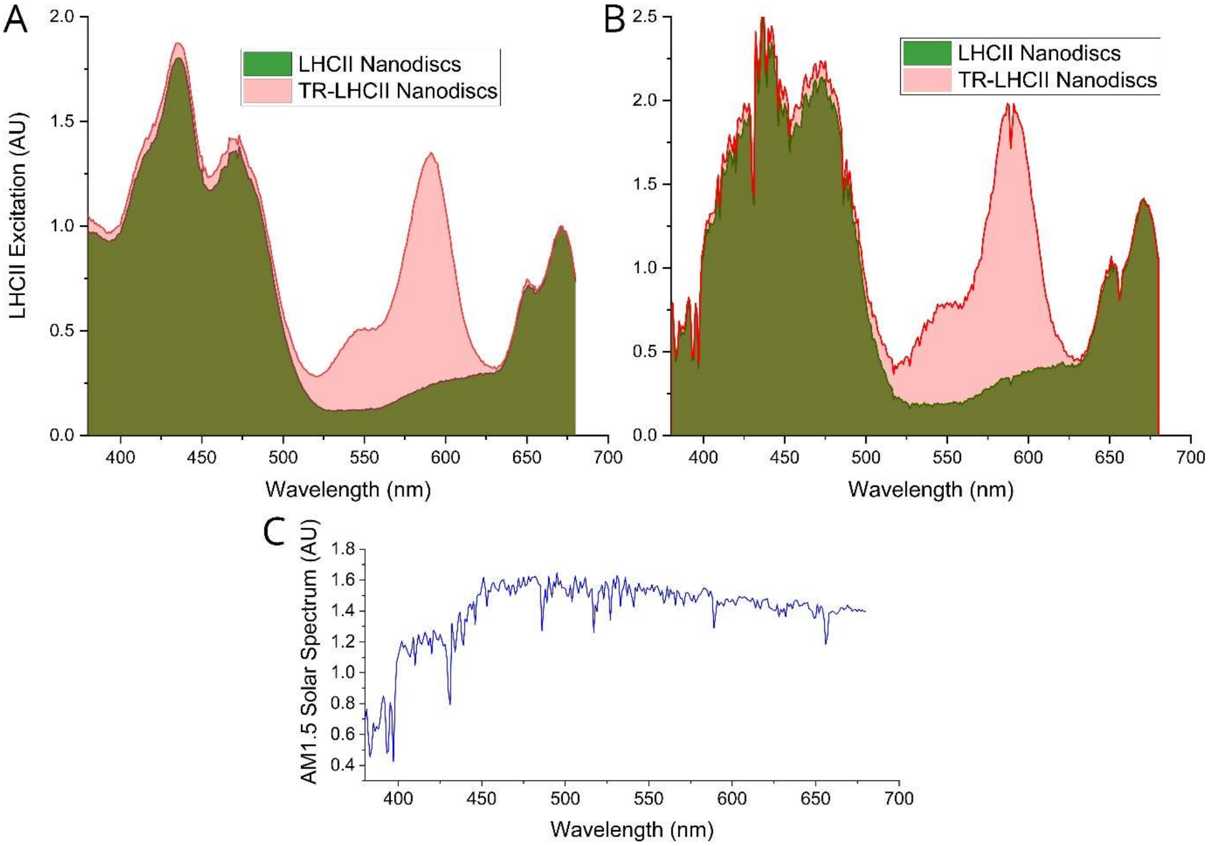


**Fig. S9.** (A) LHCII excitation spectra (emission collected at 680 nm) normalized to 1.0 at Chl a $Q_y$ peak, subsequently multiplied by the AM1.5 solar spectrum, for LHCII nanodisc and TR-LHCII nanodisc samples. (B) LHCII excitation spectra for LHCII nanodisc and TR-LHCII nanodisc samples normalised to the AM1.5 Solar spectrum. (C) AM1.5 Solar Spectrum.

LHCII nanodisc effective absorption area in the visible range 380-680 nm (*green area* in ESI Fig S9) = 277.9 AU

TR-LHCII nanodiscs effective absorption area in the visible range 380-680 nm (*red area* in ESI Fig S9) = 368.6 AU

Absorption strength (enhanced vs. original) = 368.6/277.9 = 1.326

In summary, the enhancement of LHCII in nanodiscs over the entire measurement range (the visible spectral range) was 32.6% due to the presence of TR when taking the AM1.5 solar spectrum into account. This is a higher enhancement than when all excitation wavelengths are considered equally due to the high irradiance of the AM1.5 solar spectra in the region of strong TR absorption.

## 2.14. Experimental condition for time-resolved fluorescence (TRFS) measurements.

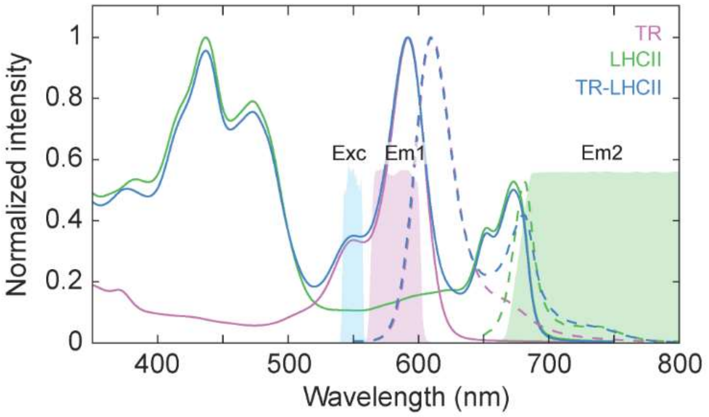


**Fig. S10.** Excitation spectrum (*Exc*) and the two emission filter ranges (*Em1* and *Em2*) employed in the TRFS measurements. The steady-state absorption (*solid lines*) and fluorescence (*dashed lines*) spectra of the samples are overlaid.

## 2.15. Emission wavelength independence of TR and LHCII fluorescence dynamics.

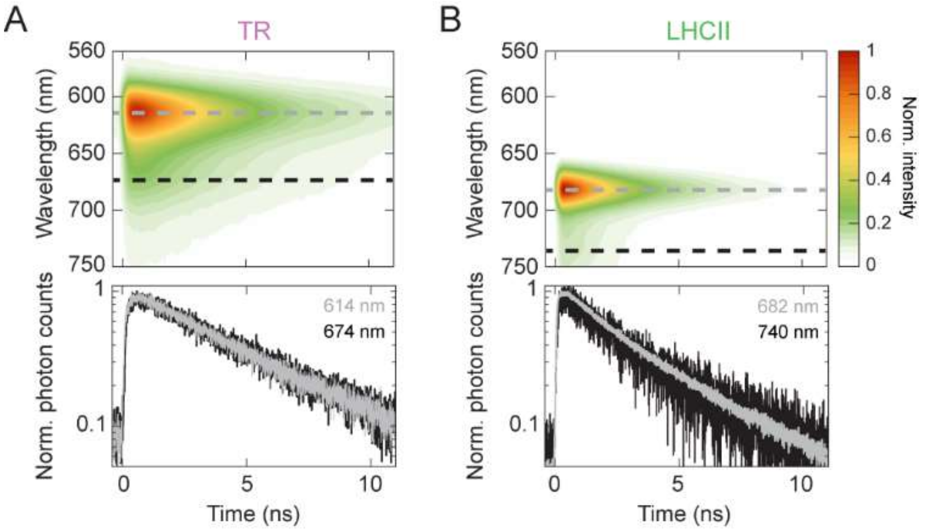


**Fig. S11.** Emission wavelength independence of TR and LHCII fluorescence decay. Top panels show the TRFS of (A) TR liposomes and (B) LHCII nanodiscs. Bottom panels show slices of the TRFS at selected emission wavelengths (indicated with dashed lines) normalized to the maximum photon count of each trace, illustrating that the fluorescence decay profiles are wavelength-independent.

## 2.16. Transient Absorption (TA) spectroscopy pump and probe properties and excitation power dependence.

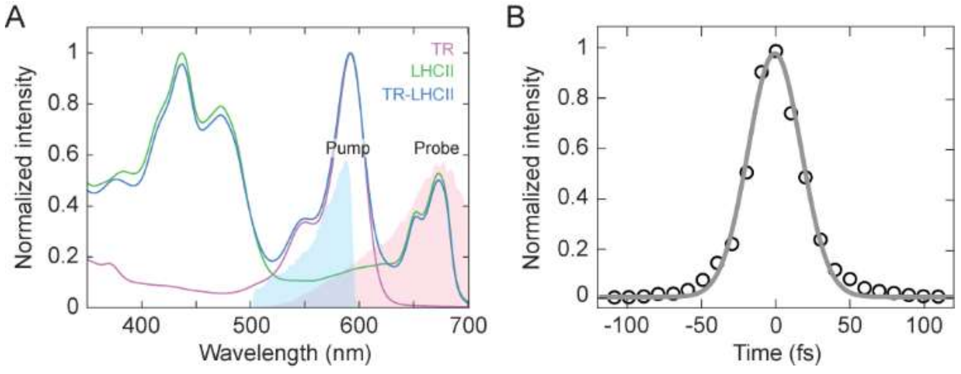


**Fig. S12.** Experimental conditions for TA measurements. (A) Pump (blue) and probe (pink) spectra overlaid with the steady-state absorption spectra of the samples. (B) Autocorrelation trace of the pump pulse. See supplementary Materials and Methods section 1.5.

**2.17. Global analysis of the TA data and decay-associated difference spectra.**

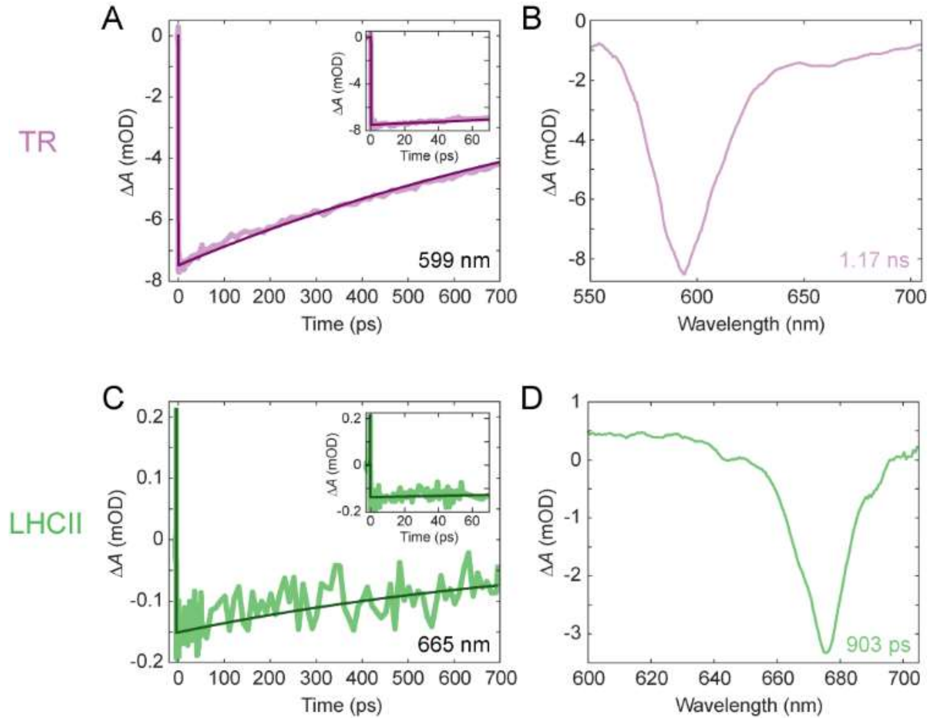


**Fig. S13.** Kinetics obtained from the TA spectroscopy data for TR liposomes and LHCII nanodiscs. Representative fit trace and the global analysis decay-associated difference spectrum (DADS) for TR liposomes (A, B) and LHCII nanodiscs (C, D). Insets in (A) and (C) show zoomed-in fit traces for the initial 70 ps. The probe wavelength of each fit trace and time constant extracted from the fit are indicated in each panel.

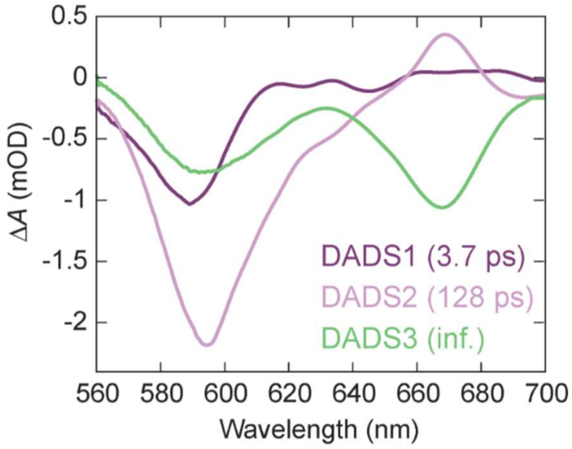


**Fig. S14.** Global analysis decay-associated difference spectra (DADS) of TR-LHCII nanodiscs sample. The time constant associated with each DADS is shown in the corresponding color. These correlate to the representative fit trace shown in main text Fig. 4A. The growth of the SE in DADS1 is less prominent than that for DADS2 due to the larger SE decay from vibronic shoulder of TR in that region at early times.

## 2.18 Global analysis of TA data for TR liposomes using a two-component fit

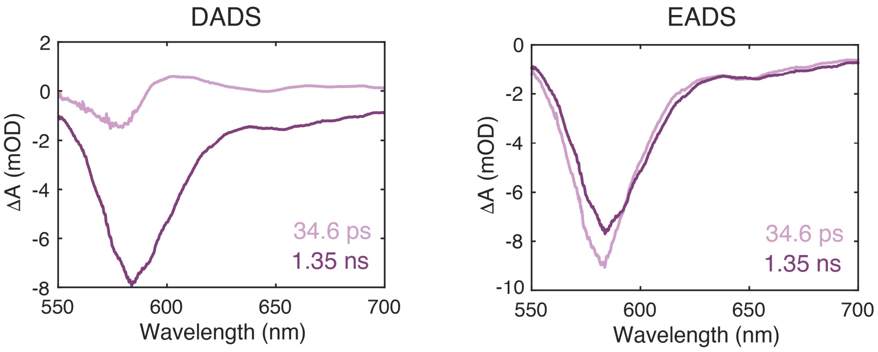


**Fig. S15.** Global analysis decay-associated difference spectra (DADS, left) and evolution-associated difference spectra (EADS, right) of TR liposomes using a two-component fit. The time constant associated with each DADS/EADS is shown in the corresponding colour.

## 2.19 Global analysis of TA data for TR-LHCII and LHCII only nanodiscs with and without 3.7 ps component

To illustrate that the 3.7 ps component is related to energy transfer, we fit the acceptor transients (675 nm) with and without the 3.7 ps component (initial 10 ps). As shown in **Fig. S16** (panel (A)), the fit fails for the TR-LHCII nanodisc sample in the absence of a 3.7 ps component, confirming that a fast rise component is present in the data. In (B) we show that the transient of the LHCII-only nanodisc control cannot be fit with a 3.7 ps component, further corroborating that the 3.7 ps component observed arises from TR-to-LHCII energy transfer.

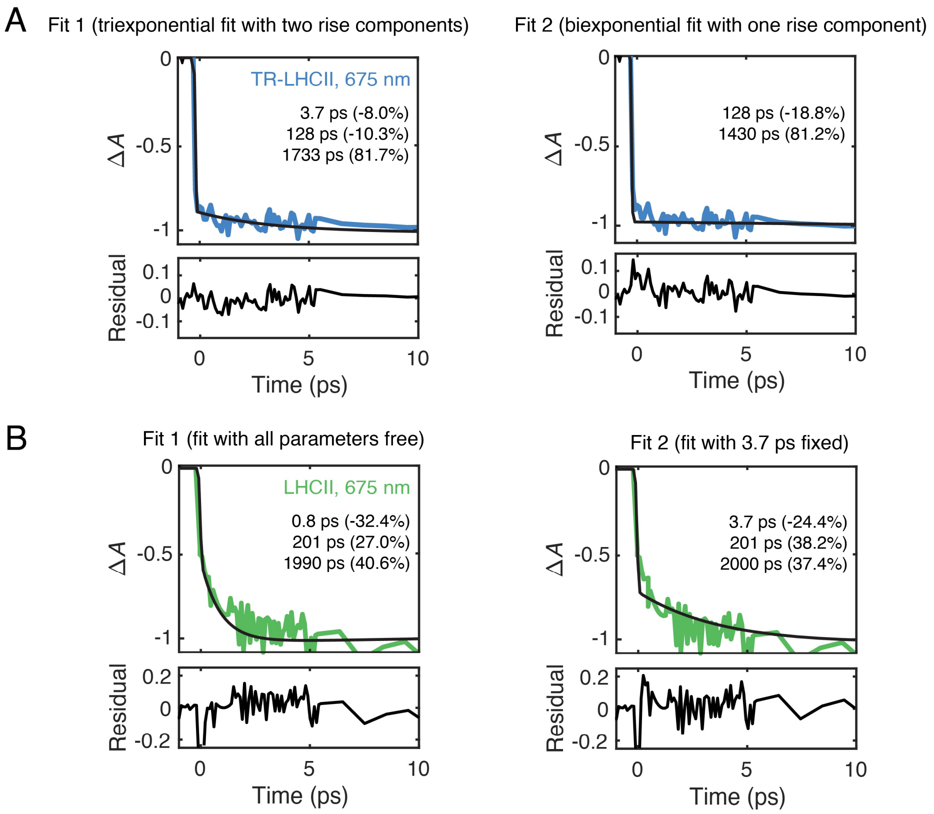


**Figure S16. Comparison of single-wavelength fit traces.** (A) TR-LHCII nanodisc probed at 675 nm. The *left panel* shows a triexponential fit (original fit shown in the main text Fig. 3H *blue trace*) with two rise components of 3.7 ps and 128 ps, the two timescales of energy transfer extracted from global analysis, and *right panel* shows a biexponential fit with only the 128 ps rise component present. (B) LHCII-only nanodisc probed at 675 nm. The *left panel* shows the best fit with all parameters freely varied (original fit shown in the main text Fig. 3H *green trace*), where a 0.8 ps rise component was extracted. The *right panel* shows a fit where the rise time constant was fixed at 3.7 ps. From the quality of the fits and structure of the residuals, we conclude that the 3.7 ps component corresponds to a fast TR-to-LHCII energy transfer step, not energy transfer among the TR molecules nor internal relaxation processes present in LHCII. All traces are normalized such that the maximum of each fit curve is –1.

## 2.20 Global analysis of TA data for TR-LHCII nanodiscs using a branched kinetic model

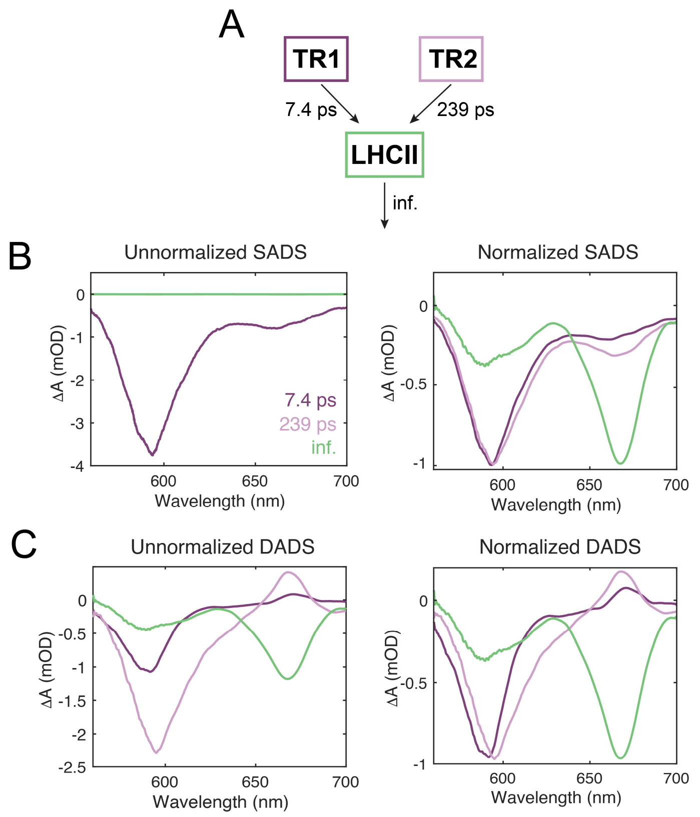


**Fig. S17:** (A) Branched kinetic model describing the energy transfer from the TR to the LHCII observed in the TA spectra. (B) Species-associated decay spectra for the three time constants extracted from target analysis and (C) the corresponding decay-associated decay spectra.

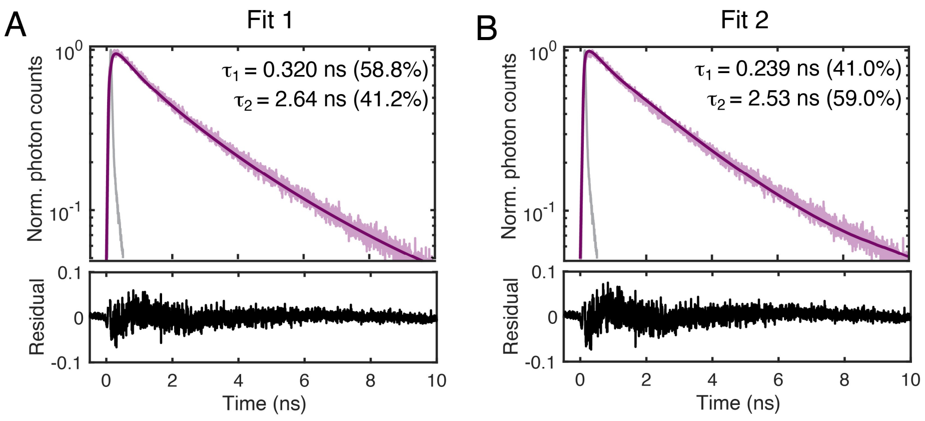


**Figure S18:** Comparison of the fits to the fluorescence decay of TR-LHCII nanodiscs when TR emission wavelength range was monitored (purple trace in Fig. 2F in the main text). (A) shows the fit result and residual when all parameters were freely varied without any constraints, as shown in Fig. 2F and Table 1, and (B) shows the fit result and residual when the first component was fixed as 0.239 ns, the time constant extracted by analysing the TA data using the branched model.

## 2.21. Expanded description of Molecular Dynamics (MD) model

The CHARMM-GUI web based interface was used to set up the MD simulation [24]. The membrane consists of 250 1,2-dioleoyl-sn-glycero-3-phosphocholine (DOPC) molecules each layer, with 37 TIP3P explicit water molecules above/under each lipid molecule. The membrane was put in a rectangular periodic boundary box with a width and breadth of 125 Å and pre-equilibrated for the protein insertion. A counter ion set of $Na^+$ and $CL^-$ was selected to neutralize the system, the final concentration was set to 0.1M to match experimental conditions. The system was first minimized under standard procedures and equilibrated over 20 ps substance volume temperature (NVT) and substance pressure temperature NPT ensembles each, an extra 20 ns NPT production run was conducted to let the system plateau.

## 2.22. Lipid diffusion constant calculation from Molecular Dynamics (MD) data.

The lipid diffusion constants can be initially derived from the MD trajectories using embedded software CPPTRAJ in AMBER. By definition, the one-dimensional diffusion coefficient *D* can be represented as:

$$\frac{\partial \varphi}{\partial t} = D\frac{\partial^2 \varphi}{\partial^2 x^2}$$

where the $\varphi$ is the concentration in dimensions of mol/$m^3$, i.e. a function that depends on both location and time. *D* is the diffusion coefficient in dimensions of $m^2$/s. This can be calculated using the Einstein relation:

$$2nD = \lim_{t\to\infty}\frac{MSD}{t}$$

where *n* is the number of dimensions, i.e., in a 2D space we have n=2 whereas in single dimension we have n=1. The *MSD* is the mean square displacement of the selected residues over the trajectory. The trajectories are first unwrapped in order to obtain the continuous path for the selected residues, otherwise the periodic boundary condition may cause severe error. Due to the fact that the diffusion coefficient is calculated from the initial position given by the trajectory, the result for small numbers of atoms will be inherently stochastic for small number of sampled atoms. To deal with this we averaged the result over the 5 MD trajectories and used all the selected residues in the system to calculate its diffusion coefficient. The result is shown below in Table S3:

**Table S3.** Diffusion constants for the common lipid 1,2-dioleoyl-*sn*-glycero-3-phosphocholine (DOPC) and Texas Red calculated in MD simulations

| Lipid type | Diffusion constant calculated from our MD data (μ$m^2$/s) | Literature value (μ$m^2$/s) from [25] |
|---|---|---|
| DOPC | 8.4 | 5 to 14 |
| Texas Red DHPE | 4.5 | N/A |

**2.23. Pigment cofactor positions in the LHCII monomer from MD data.**
The 50-ns-long simulation did not reveal any significant displacement of the pigment cofactors in the LHCII monomer (as shown in **Fig. S19A-C**). The pigments generally remain their initial position in the crystal structure adopted from PDB structure 1RWT. The clusters of chlorophylls (Chl) on the lumen side are less efficient in terms of energy transfer compared to the stromal side, as they are more separated in space. The subtle changes to pigment positions which do occur are briefly discussed below.

5 out of the 6 Chl *b* molecules surround the helix C at the interface between the monomers, with the remaining Chl *b* 608 left of the lutein pair, as shown in **Fig. S19D**. This region is proposed to be critical in terms of energy transfer between the monomers. Now considering the Chl *a* molecules, the "terminal emitter" generally remains in its initial position, with Chl *a* 611 slightly displaced towards Chl *b* 608. This movement is a result of monomerizing the LHCII trimer which leave Chlb601 highly lipid exposed. It has been shown that monomerization of LHCII does lead to significant changes in the Chl b region of the absorption spectrum, implying changes in pigment position and orientation [26]. In previous MD studies this movement can be prevent by imposing positional constraints during the equilibration phase [27]. However, we omitted this step as Chlb601 was not one of the pigments considered when we calculated couplings. Moreover, its movement had a negligible effect on the pigments that we did consider. **Fig. S19E** shows the positions of the luteins and Chl *a* molecules.

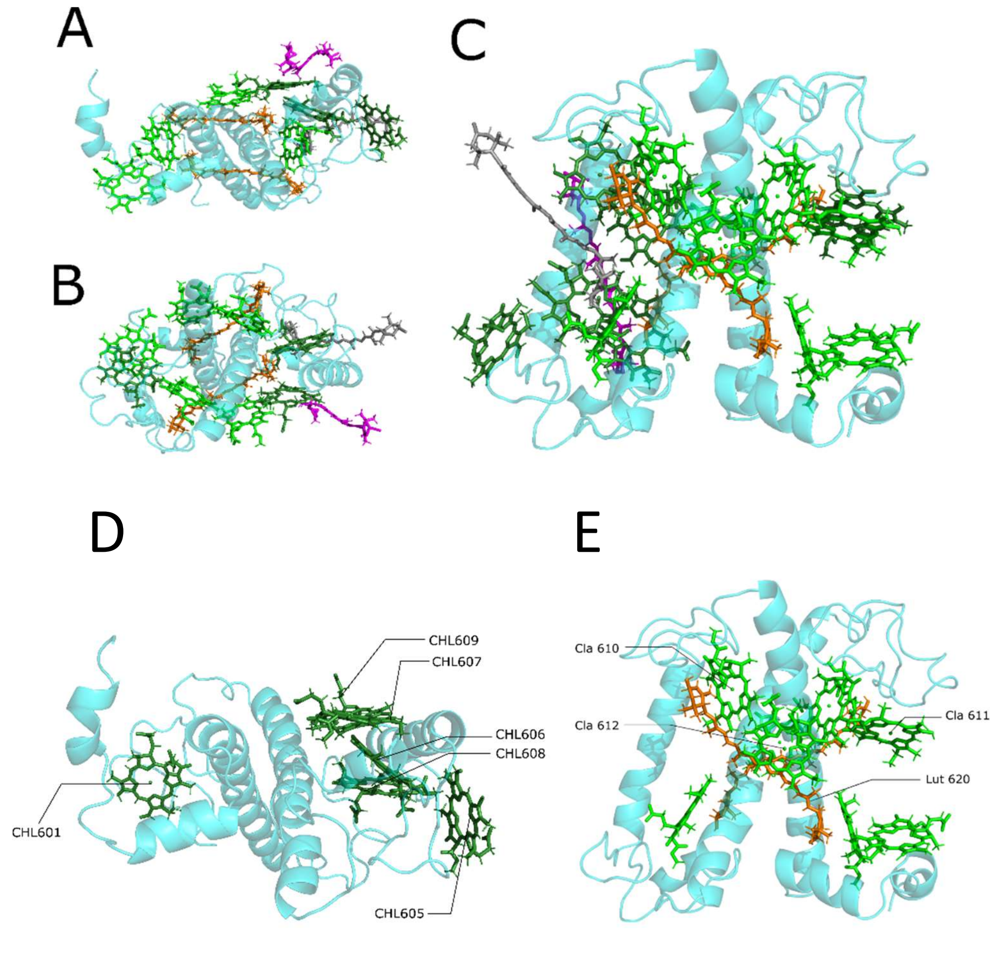


**Fig. S19.** Various views of the LHCII monomer from our MD simulations. (A) The top view (stromal side), (B) bottom view (luminal side) and (C) the side view of the pigment

representation. Chlorophyll *a* coloured in *light green*; chlorophyll *b* coloured in *dark green*; luteins coloured in *orange*; neoxanthin and violaxanthin coloured in *magenta* and *grey*, respectively. The chlorophyll tails are hidden for clarity. (D) Representation of chlorophyll *b* in the LHCII monomer (top view). (E) Representation of chlorophyll *a* molecules and the lutein molecules in the LHCII monomer (side view).

**2.24. Calculations for the rate of energy transfer and Förster radius.**

The timescale for energy transfer from TR to the LHCII monomer is characterized by the following rate constant:

$$\boldsymbol{k_{LHCII\leftarrow TR}} = \sum_{i} \boldsymbol{k_{m\leftarrow TR}} \quad \textbf{(1)}$$

where $k_{m\leftarrow TR}$ represents the rate of transfer to individual states within LHCII. Formally these states should be delocalized exciton states that arise due to couplings between the chlorophylls (Chls). However, it has been shown that these couplings result in minimal peak shifts and redistribution of oscillator strength relative to the uncoupled state [28]. Therefore, in our approximate model, $k_{m\leftarrow TR}$ represent pigment-to-pigment transfer rates. The summation should run over all Chl *a* and Chl *b* pigments inside the monomer. However, transfer to the inner pigments is likely to be significantly reduced due to screening by the protein and outer pigments. We therefore assume that transfer occurs predominantly to the pigments close to the membrane-exposed part of LHCII. The validity of this assumption may be reduced for very close separations between TR and LHCII. We note that energy transfer to the Chls can, in principle, occur through one of two transitions, either the strongly absorbing $Q_y$ or the higher-energy but less absorbing $Q_x$ transition. The total pigment-to-pigment rate will be the sum of these two pathways,

$$\boldsymbol{k_{m\leftarrow TR}} = \boldsymbol{k_{Q_y\leftarrow TR}} + \boldsymbol{k_{Q_x\leftarrow TR}} \quad \textbf{(2)}$$

The individual rates are calculated according to the Fermi Golden Rule,

$$\boldsymbol{k_{Q_{x/y}\leftarrow TR}} = \boldsymbol{2\pi\left|J_{m,TR}\right|^2 \int_{-\infty}^{\infty} d\omega\, F_{TR}(\omega) A_{Q_{x/y}}(\omega)} \quad \textbf{(3)}$$

where $J_{Q_{x/y},TR}$ is the resonance coupling between the electronic transitions of the donor (TR) and acceptor (Chl), $F_{TR}(\omega)$ is the fluorescence spectrum of the donor and $A_{Q_{x/y}}(\omega)$ is the absorption spectrum of the acceptor (with all spectra in the frequency domain).

The resonance coupling is the Coulomb interaction between electronic transition densities on the two molecules. So long as the separation between pigments is larger than the length-scale of each molecule then these transition densities can be approximated as point transition dipole moments located at their centre of charge. For the Chls this is approximately at the central magnesium of the tetrapyrrole head while for TR this is assumed to be at the middle of the central ring [29]. The dipole-dipole coupling is,

$$\boldsymbol{J_{Q_{x/y},TR}} \approx \frac{\boldsymbol{\kappa_{Q_{x/y},TR}\left|\mu_{Q_{x/y}}\right|\left|\mu_{TR}\right|}}{\boldsymbol{4\pi\varepsilon_0\left|R_{Q_{x/y},TR}\right|^3}} \quad \textbf{(4)}$$

where $\left|\mu_{Q_{x/y}}\right|$ and $|\mu_{TR}|$ are the magnitudes of the two transition dipole moments, $R_{m,TR}$ is their relative separation distance and $\kappa_{m,TR}$ describes their relative orientation,

$$\kappa_{m,TR} = \hat{\boldsymbol{\mu}}_{Q_{x/y}} \cdot \hat{\boldsymbol{\mu}}_{TR} - 3\left(\hat{\boldsymbol{\mu}}_{Q_{\frac{x}{y}}} \cdot \hat{\mathbf{R}}_{Q_{\frac{x}{y}},TR}\right)\left(\hat{\boldsymbol{\mu}}_{TR} \cdot \hat{\mathbf{R}}_{Q_{\frac{x}{y}},TR}\right) \quad \textbf{(5)}$$

where $\hat{\boldsymbol{\mu}}_{Q_{x/y}}$, $\hat{\boldsymbol{\mu}}_{TR}$ and $\hat{\mathbf{R}}_{m,TR}$ are unit vectors.

The pigment-to-pigment separation distances are read directly from the uncorrelated snapshots of the MD trajectories. The transition dipoles for the $Q_y$ transitions of Chl *a* and Chl *b* are given lengths of 4.5 Debye and 3.6 Debye, respectively, and they are assumed to approximately lie along a line connecting two opposite nitrogen atoms in the tetrapyrrole ring [30]. The $Q_x$

dipoles are assumed to be 0.67 the length of the $Q_y$ dipoles and lie in an orthogonal direction in the tetrapyrrole plane [31]. The dipole moment of TR has been reported to lie along the 5-ring chromophore [29]. We assumed a representative dipole length of 10 Debye to be consistent with the 7-13 Debye values reported for various rhodamine derivatives [32].

To evaluate the spectral overlap integral in Eqn. (3) we need spectra for both TR and the $Q_{x/y}$ transitions of Chl *a* and *b*. For TR we simply use the experimental fluorescence spectrum (see **Fig. S20**, *light blue*). This consists of a single broad peak with an almost featureless vibronic satellite. It is not straightforward to extract the $Q_x$ and $Q_y$ contributions of an experimentally-measured absorption spectrum of Chl *a* or Chl *b*. Therefore, we instead use a well-known ansatz function to describe each of them [33]. The absorption spectrum in the frequency domain is the Fourier transform of the first-order response function,

$$A_{Q_{x/y}}(t) = \exp\left(-i\omega_{Q_{x/y}}t - g_{Q_{x/y}}(t)\right) \quad (6)$$

where $\omega_{Q_{x/y}}$ is the electronic transition frequency (i.e., the position of the 0-0 absorption peak) and $g_{Q_{x/y}}(t)$ is the complex *line-broadening function*. The latter characterizes the coupling between the electronic transition and the nuclear vibrations of the pigment (which define the broadening and vibronic structure of the spectrum). These nuclear vibrations are stochastic in nature and enter the line-broadening function by their spectral density, $C''_{Q_{x/y}}(\omega)$,

$$g_{Q_{x/y}}(t) = \int_0^\infty \frac{d\omega}{\pi\omega^2} C''_{Q_{x/y}}(\omega) \left[(1-\cos(\omega t))\coth\left(\frac{\hbar\omega}{2k_B T}\right) + i(\sin(\omega t) - \omega t)\right] \quad (7)$$

The spectral density is essentially the spectrum of molecular vibrational frequencies, $\omega$, weighted by their relative coupling to the electronic transition energy. We use the familiar over-damped Brownian oscillator ansatz (also known as the Drude model),

$$C''_{Q_{x/y}}(\omega) = 2\lambda \frac{\gamma\omega}{\omega^2 + \gamma^2} \quad (8)$$

where $\lambda$ is the vibrational reorganization energy associated with the transition and $\gamma$ is the damping frequency of the vibrations. We use standard values of $\lambda \sim 40\ \mathrm{cm}^{-1}$ and $\gamma^{-1} = 53\ fs$ [9]. We also assume that $\omega_{Q_y} = 14{,}900\ \mathrm{cm}^{-1}$ and $15{,}300\ \mathrm{cm}^{-1}$ for Chl *a* and *b* respectively with $\omega_{Q_x} = \omega_{Q_y} + 1000\ \mathrm{cm}^{-1}$ for both. These calculations yield estimated line shapes for the $Q_x$ and $Q_y$ transitions of Chl *a* and *b* shown in **Fig. S20** (*orange, grey, yellow, dark blue*). These line shapes can be combined at a $Q_x/\ Q_y$ ratio of 0.45 to reproduce the LHCII absorption spectra as shown in **Fig. S21**.

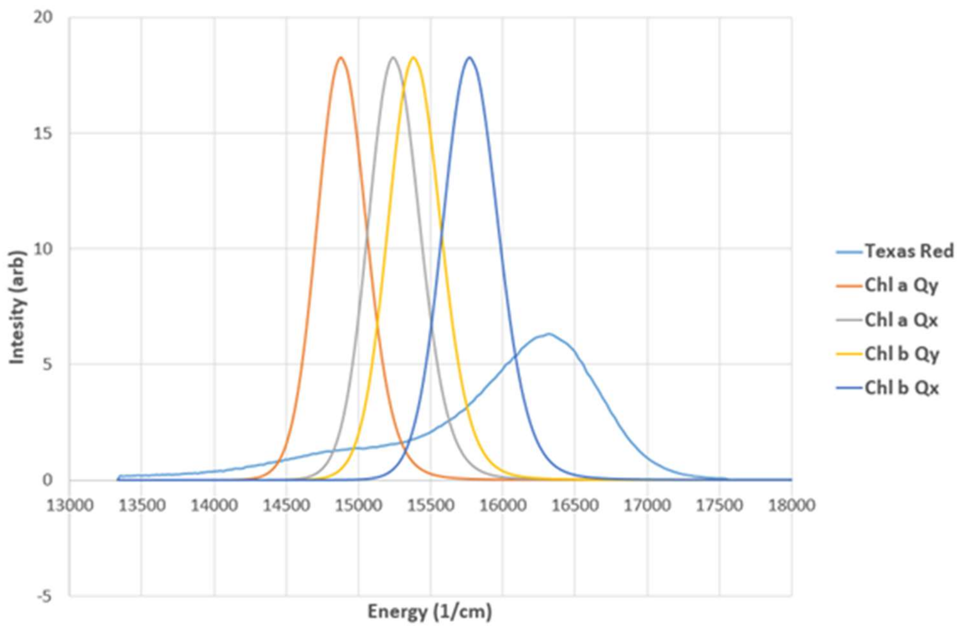


**Fig. S20.** The normalized line shapes for Chl $a$ and $b$ are shown with the normalized TR fluorescence spectrum. The relative intensities of the actual transitions are captured within the resonance couplings. Therefore, the relative heights of each peak shown here have no physical meaning beyond being determined by the normalization condition. The TR spectrum is experimental data acquired on a sample of TR liposomes (a fluorescence emission spectrum with excitation at 560 nm, converted from being intensity as a function of wavelength to intensity as a function of energy).

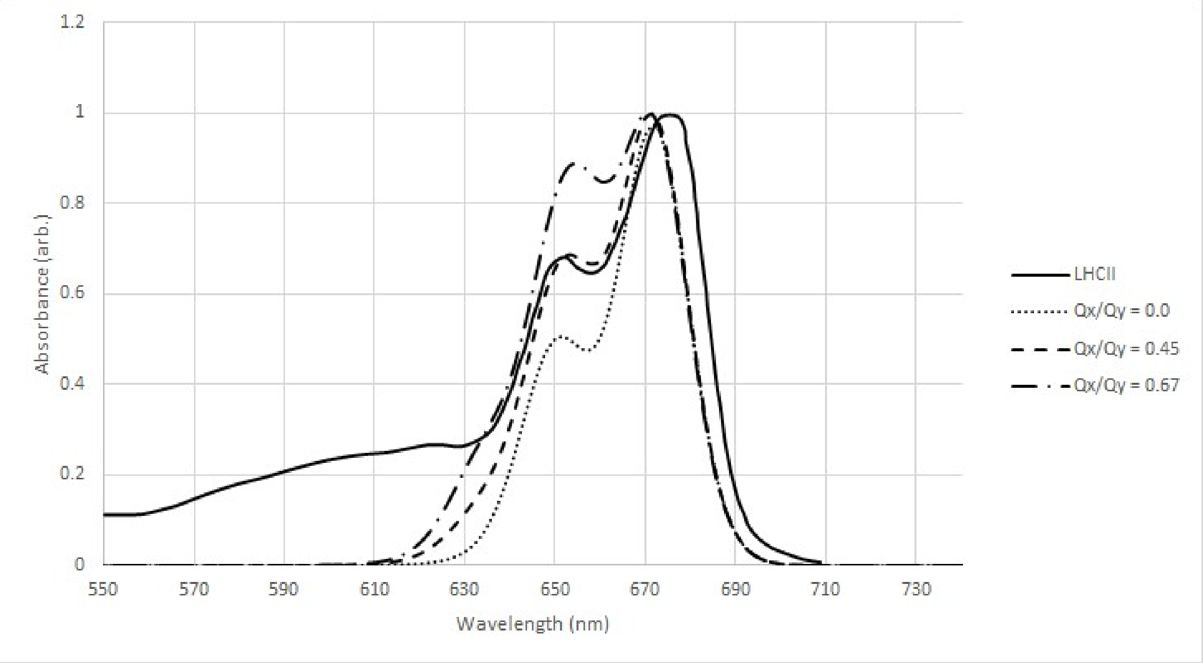


**Figure S21**: Reconstruction of the LHCII absorption spectrum from $Q_x$ and $Q_y$ line shapes. The experimentally-measured spectrum for LHCII is shown as a solid line, the reconstructed spectrum with varying $Q_x$/ $Q_y$ ratios are shown with dashed lines (ratios as indicated in the figure legend).

**2.25. Transition dipole moment for Texas Red.**

During the process calculating the coupling interaction between the chlorophyll and the Texas Red molecules, we took the approximation that this can be simplified to a point-dipole problem. Here we define the Chl *a* $Q_y/Q_x$ transition dipole moments as a vector from its centrally bound Mg atom to the NB/NC atoms respectively (*black arrow* and *opaque arrow* in **Fig. S22**). Note that the real dipole moment of the $Q_y$ transition originates from the Mg atom to the NB atom but slightly deviated towards the NC atom. This is believed to have little effect given the distance between the pair and the fluctuations along the trajectory. We took the convenience that the $Q_x$ transition dipole moment is roughly orthogonal to the $Q_y$ and thus pointing to the NC atom. The transition dipole strength of Chl *a* was set to 4.5 Debye. The same direction of the dipole moments was applied to Chl *b* molecules, with the strength of 3.8 Debye. For the Texas Red molecule, the transition dipole moment was approximated from the middle point of C20 and C24 to the middle point of C31 and C25 (shown in black arrow in the figure below). The dipole strength is set to 10 Debye.

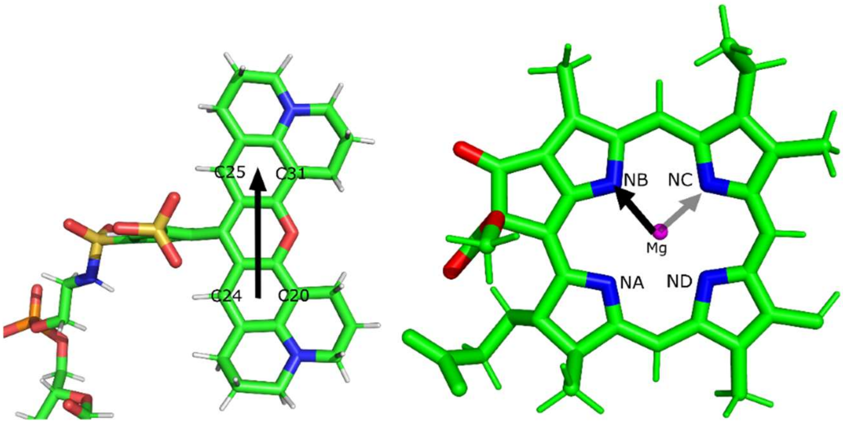


**Fig. S22.** Transition dipole moment of the Texas Red (*left*) and chlorophyll *a* molecules (*right*). Approximated dipole moments are represented as arrows in figure.

## 3. Supplementary References


1. Adams, P.G., et al., *Correlated fluorescence quenching and topographic mapping of Light-Harvesting Complex II within surface-assembled aggregates and lipid bilayers.* Biochim Biophys Acta, 2018. **1859**(10): p. 1075-1085.
2. Son, M., et al., *Observation of dissipative chlorophyll-to-carotenoid energy transfer in light-harvesting complex II in membrane nanodiscs.* Nat Commun, 2020. **11**(1): p. 1295.
3. Adams, P.G., et al., *Exploiting lipopolysaccharide-induced deformation of lipid bilayers to modify membrane composition and generate two-dimensional geometric membrane array patterns.* Sci. Rep., 2015. **5**: p. 10331.
4. Hancock, A.M., et al., *Proteoliposomes as energy transferring nanomaterials: enhancing the spectral range of light-harvesting proteins using lipid-linked chromophores.* Nanoscale, 2019. **11**(35): p. 16284-16292.
5. Yuan, P. and D. Walt, *Calculation for Fluorescence Modulation by Absorbing Species and Its Application to Measurements Using Optical Fibers.* Analytical Chemistrty, 1957. **59**: p. 2391-2394.
6. Perri, A., et al., *Time- and frequency-resolved fluorescence with a single TCSPC detector via a Fourier-transform approach.* Opt Express, 2018. **26**(3): p. 2270-2279.
7. Luchowski, R., et al., *Instrument response standard in time-resolved fluorescence.* Rev Sci Instrum, 2009. **80**(3): p. 033109.
8. Son, M., S. Mosquera-Vazquez, and G.S. Schlau-Cohen, *Ultrabroadband 2D electronic spectroscopy with high-speed, shot-to-shot detection.* Opt Express, 2017. **25**(16): p. 18950-18962.
9. Reimers, J.R., et al., *Assignment of the Q-bands of the chlorophylls: coherence loss via Qx - Qy mixing.* Sci Rep, 2013. **3**: p. 2761.
10. Duan, H.G., et al., *Two-Dimensional Electronic Spectroscopy of Light-Harvesting Complex II at Ambient Temperature: A Joint Experimental and Theoretical Study.* J Phys Chem B, 2015. **119**(36): p. 12017-27.
11. Snellenburg, J.J., et al., *Glotaran: A Java-Based Graphical User Interface for the R Package TIMP.* Journal of Statistical Software, 2012. **49**(3): p. 1--22.
12. van Stokkum, I.H., D.S. Larsen, and R. van Grondelle, *Global and target analysis of time-resolved spectra.* Biochim Biophys Acta, 2004. **1657**(2-3): p. 82-104.
13. Blanchette, C.D., et al., *Quantifying size distributions of nanolipoprotein particles with single-particle analysis and molecular dynamic simulations.* J Lipid Res, 2008. **49**(7): p. 1420-30.
14. Blanchette, C.D., et al., *Atomic force microscopy differentiates discrete size distributions between membrane protein containing and empty nanolipoprotein particles.* Biochim Biophys Acta, 2009. **1788**(3): p. 724-31.
15. Natali, A., et al., *Light-harvesting Complexes (LHCs) Cluster Spontaneously in Membrane Environment Leading to Shortening of Their Excited State Lifetimes.* J Biol Chem, 2016. **291**(32): p. 16730-9.
16. Titus, J.A., et al., *Texas red, a hydrophilic, red-emitting flourophore for use with flourescein in dual parameter flow microfluorometric and fluorescence microscopic studies.* Journal of Immunological Methods, 1982. **50**(2): p. 193-204.

17. Tutkus, M., et al., *Fluorescence Microscopy of Single Liposomes with Incorporated Pigment-Proteins.* Langmuir, 2018. **34**(47): p. 14410-14418.

18. Blankenship, R.E., *Molecular Mechanisms of Photosynthesis.* 2014(Second Edition).

19. Förster, T., *Delocalized excitation and excitation transfer.* Modern Quantum Chemistry Istanbul Lectures, 1965. **3**: p. 93-137.

20. van Eerden, F.J., et al., *Characterization of thylakoid lipid membranes from cyanobacteria and higher plants by molecular dynamics simulations.* Biochimica et Biophysica Acta (BBA) - Biomembranes, 2015. **1848**(6): p. 1319-1330.

21. Goral, T.K., et al., *Light-harvesting antenna composition controls the macrostructure and dynamics of thylakoid membranes in Arabidopsis.* Plant J, 2012. **69**(2): p. 289-301.

22. Hink, M.A., et al., *Practical Use of Corrected Fluorescence Excitation and Emission Spectra of Fluorescent Proteins in Förster Resonance Energy Transfer (FRET) Studies.* Journal of Fluorescence, 2003. **13**(2): p. 185-188.

23. Visser, A.J., et al., *Time-resolved FRET fluorescence spectroscopy of visible fluorescent protein pairs.* Eur Biophys J, 2010(1432-1017 (Electronic)).

24. Jo, S., et al., *CHARMM-GUI: A web-based graphical user interface for CHARMM.* Journal of Computational Chemistry, 2008. **29**(11): p. 1859-1865.

25. Oradd, G., V. Shahedi, and G. Lindblom, *Effect of sterol structure on the bending rigidity of lipid membranes: A (2)H NMR transverse relaxation study.* Biochim Biophys Acta, 2009. **1788**(9): p. 1762-71.

26. Gradinaru, C.C., et al., *The Flow of Excitation Energy in LHCII Monomers: Implications for the Structural Model of the Major Plant Antenna.* 1998. **75**(6): p. 3064-3077.

27. Papadatos, S., A.C. Charalambous, and V. Daskalakis, *A pathway for protective quenching in antenna proteins of Photosystem II.* Scientific Reports, 2017. **7**(1).

28. Balevicius, V. and C.D.P. Duffy, *Excitation quenching in chlorophyll-carotenoid antenna systems: 'coherent' or 'incoherent'.* Photosynth Res, 2020. **144**(3): p. 301-315.

29. Skaug, M.J., M.L. Longo, and R. Faller, *Computational Studies of Texas Red−1,2-Dihexadecanoyl-sn-glycero-3-phosphoethanolamine—Model Building and Applications.* The Journal of Physical Chemistry B, 2009. **113**(25): p. 8758-8766.

30. Knox, R.S. and B.Q. Spring, *Dipole Strengths in the Chlorophylls ¶†.* Photochemistry and Photobiology, 2003. **77**(5): p. 497-501.

31. Damjanović, A., T. Ritz, and K. Schulten, *Excitation transfer in the peridinin-chlorophyll-protein of Amphidinium carterae.* Biophysical journal, 2000. **79**(4): p. 1695-1705.

32. Chung, P.-H., C. Tregidgo, and K. Suhling, *Determining a fluorophore's transition dipole moment from fluorescence lifetime measurements in solvents of varying refractive index.* Methods and Applications in Fluorescence, 2016. **4**(4): p. 045001.

33. Valkunas, L., D. Abramavicius, and T. Mancal, *Molecular Excitation Dynamics and Relaxation: Quantum Theory and Spectroscopy*. 2013.

34. ASTM G173-03(2020), Standard Tables for Reference Solar Spectral Irradiances: Direct Normal and Hemispherical on 37° Tilted Surface, ASTM International, West Conshohocken, PA, 2020, www.astm.org
